# FedSwin-LHTP: Structure-Aware Hessian-Inspired Token Pruning for Efficient Federated Skin Lesion Classification

**DOI:** 10.3390/diagnostics16162637

**Published:** 2026-08-19

**Authors:** Muhammad Awais, Riaz Hussain Junejo

**Affiliations:** 1 Department of Computer Science, College of Computer, Qassim University, Buraydah 52571, Saudi Arabia; 2 Department of Computer Engineering, COMSATS University Islamabad, Wah Campus, Wah Cantt 47040, Pakistan; riazjunejo@ciitwah.edu.pk

**Keywords:** deep learning, federated learning, vision transformers, computer vision, optimization, AI, medical imaging

## Abstract

**Background:** Skin cancer encompasses a diverse range of malignancies and remains a significant global health challenge. Accurate machine-learning-assisted diagnosis can substantially improve patient outcomes through early detection and timely clinical intervention. Federated Learning (FL) enables privacy-preserving collaborative model training across multiple healthcare institutions while ensuring that sensitive patient data remain decentralized. However, deploying advanced architectures such as Vision Transformers (ViTs) in clinical environments is challenging due to the high computational demands of self-attention mechanisms. **Methods:** This work proposes FedSwin-LHTP, an efficient federated learning framework for skin lesion classification that integrates a Swin Transformer backbone with a Lightweight Hessian-Inspired Token Pruning (LHTP) mechanism. LHTP estimates token importance using a second-order Taylor approximation around converged local model parameters to identify less informative patch tokens, enabling the early pruning of redundant representations without explicitly computing the Hessian matrix. Furthermore, the framework incorporates the FedProx optimization objective to mitigate client drift under heterogeneous non-IID data distributions. The proposed framework is evaluated on the HAM10000 and ISIC datasets under realistic non-IID federated settings. **Results:** Experimental results demonstrate stable convergence, effective knowledge aggregation, and robust diagnostic discrimination across distributed clients. By adaptively pruning approximately 60% of Stage-1 tokens, the proposed framework substantially reduces the computational burden of local transformer processing while maintaining high multiclass classification performance, achieving an accuracy of up to 96.1% on the evaluated datasets. **Conclusions:** These results highlight the potential of FedSwin-LHTP as a practical, privacy-preserving, and resource-efficient solution for collaborative healthcare intelligence.

## 1. Introduction

Skin cancer is among the most common forms of cancer worldwide, posing a significant health challenge. It includes a wide range of skin lesions, from benign conditions such as melanocytic nevi and keratoses to precancerous abnormalities like actinic keratosis, as well as malignant cancers including melanoma, basal cell carcinoma, and squamous cell carcinoma. Because treatment success is closely linked to the stage at which the disease is detected, early and accurate diagnosis plays a crucial role in improving patient outcomes. For example, patients diagnosed with localized melanoma at an early stage have a five-year survival rate exceeding 99%, whereas survival decreases substantially once the cancer spreads to distant organs [1]. As a result, machine-learning-based diagnostic systems are attracting increasing attention as supportive tools for clinicians in the detection and assessment of skin cancer.

Recently, deep learning has shown substantial potential for a wide variety of biomedical classification tasks. However, the lack of well-annotated datasets, especially for rare disease classes and the strict laws for patient privacy such as HIPAA [2] and GDPR [3] that disallow centralized aggregation of medical data, makes skin lesion classification challenging. The classification job is further complicated by differences in the visual features of skin lesions, intra-class variability, and inter-class similarity. Poor model generalization brought on by differences in imaging procedures, acquisition tools, and institutional data distributions throughout healthcare settings further reduces diagnostic accuracy.

Federated Learning, an emerging paradigm, offers a privacy-preserving solution to the data-silo problem by enabling decentralized and collaborative training of deep learning models across multi-institutional datasets without sharing sensitive patient data [4]. In a typical FL network, a central-aggregation server initializes and distributes a global model to multiple participating clients, each representing a geographically distributed healthcare institution with its own clinical specialization, patient population, and imaging equipment. In such a heterogeneous environment, the suitable choice of the deep learning model and its training are critical for the overall performance. Deep convolutional neural networks (CNNs) have demonstrated good performance for different applications, including biomedical image analysis [5,6]. However, CNNs mostly focus on local feature extraction and their effective receptive field often fails to cover long-range dependency between distant regions of an image. This limitation is important in skin lesion analysis, because diagnostically relevant patterns can be found in distant regions of an image. Expanding the receptive field through larger kernels or deeper architectures typically increases computational complexity and may compromise fine-grained spatial information [7].

Vision Transformers [8] are based on self-attention operations to capture long-range spatial relationships across the entire image. However, ViTs do not have the inductive biases of translation equivariance and locality, which CNNs have naturally, and hence, require orders of magnitude more training data to perform on par with CNNs. This data requirement is particularly problematic in federated settings where data are distributed across multiple institutions and are often scarce, severely imbalanced and heterogeneous in nature. Meanwhile, the self-attention mechanism of ViTs has a quadratic complexity in the number of image tokens *N* [9], which requires a large memory and computation. These requirements may limit deployment on resource-constrained clinical devices, which are common in real-world healthcare settings. To improve efficiency, several token pruning methods are proposed, including static and dynamic image-dependent strategies [10,11]. However, these methods are primarily designed for centralized training and do not account for the heterogeneous (non-IID) data distributions and decentralized optimization inherent in FL. Consequently, they produce inconsistent token selection across clients, leading to unstable model aggregation and degraded global performance. Token pruning is fundamentally a resource–accuracy trade-off. By removing tokens, we reduce computational burden but risk discarding information essential for accurate classification. The challenge lies in determining which tokens to remove and how many can be safely pruned without compromising diagnostic performance. More recently, population-based metaheuristic optimization techniques have shown potential for adaptive token selection in federated environments. In our previous work [12], a Quantum-Inspired Evolutionary Algorithm was used to identify informative tokens, achieving reduced computational complexity while maintaining strong predictive performance.

Swin Transformer [13], a hierarchical ViT variant that performs a shifted window-based self-attention to reduce computational complexity while maintaining strong representational capability. However, Swin Transformer still processes a large number of tokens in multiple stages, leading to significant memory and computational burden. To address this challenge, this work proposes FedSwin-LHTP, a federated skin lesion classification framework that incorporates a lightweight Hessian-inspired token pruning strategy within a Swin Transformer backbone. Token pruning represents an inherent tradeoff between computational efficiency and predictive accuracy: overly aggressive pruning may discard diagnostically important information while overly conservative pruning provides limited computational savings. To this end, the proposed framework employs an adaptive pruning strategy that can keep the most informative tokens and remove the redundant ones selectively. Our method identifies less informative patch tokens using a low-cost second-order Taylor approximation using converged local model parameters, while the traditional Hessian-based methods explicitly compute the full Hessian matrix to evaluate the importance of many transformer tokens [14]. Moreover, we propose a structure-aware pruning strategy to preserve the local attention consistency in Swin Transformer windows, so that the important spatial relationship can be preserved in token reduction. In addition, to improve training stability in the presence of heterogeneous client data distributions, the proposed framework incorporates FedProx-based proximal regularization, which helps reduce client drift and supports more stable global model convergence during federated learning. Extensive simulations on the HAM10000 and ISIC2019 benchmark datasets under realistic non-IID data distributions demonstrate the effectiveness and the numerical stability of the proposed approach. These datasets contain a wide range of benign and malignant skin lesion classes like melanocytic nevus (NV), melanoma (MEL), basal cell carcinoma (BCC), benign keratosis-like lesions (BKL), actinic keratosis (AK/AKIEC), dermatofibroma (DF), vascular lesions (VASC) and squamous cell carcinoma (SCC). However, both datasets suffer from severe class imbalance and high intra-class variability, leading to biasing the model towards the majority classes, lowering the generalization of rare lesions and increasing the feature overlap between visually similar categories. To address these challenges, the proposed framework employs weighted cross-entropy loss to emphasize minority classes, FedProx regularization to stabilize training under heterogeneous non-IID data distributions, and adaptive LHTP pruning to preserve diagnostically relevant features irrespective of class prevalence.

## 2. Literature Review

The current landscape of FL frameworks applied to dermatological image analysis can be broadly classified into two methodological tracks: (1) CNN-based FL paradigms and (2) ViT-based decentralized architectures. Recent research efforts in these domains are mostly concentrated on local optimization to reduce computation and parameter overhead and privacy-preserving regularizers to improve collaborative learning. Table 1 presents a comprehensive summary of some recent works targeting decentralized skin lesion classification. These works are discussed category-wise as in the following sub-sections.

### 2.1. CNN-Based Federated Learning Approaches

The early development of FL frameworks for collaborative skin lesion classification was primarily based on CNNs, because their inherent inductive biases, including translation equivariance, locality, and weight sharing, enable effective learning from limited medical datasets without requiring massive training corpora. Furthermore, their relatively low computational complexity makes them well suited for deployment on resource-constrained clinical edge devices. To further improve efficiency, researchers adopted lightweight architectures such as EfficientNetV2S and MobileNetV2, which employ depthwise separable convolutions, squeeze-and-excitation blocks, and compound scaling to reduce model parameters and computational cost while maintaining competitive diagnostic performance. However, CNN-based FL models often suffer from the performance degradation under non-IID data distributions due to client drift, where local models diverge from the global objective as each client optimizes on its own data distribution. This divergence causes inconsistent gradients, bias to locally dominant lesion classes, and lower generalization to under-represented lesion classes after model aggregation.

Early studies employed either custom CNN architectures or locally fine-tuned pretrained backbones at participating institutions. For example, ref. [15] developed a custom FL-CNN for four-class skin lesion classification and achieved an accuracy of 94%. To improve deployment in resource-constrained environments, ref. [16] trained EfficientNetV2S, EfficientNetB3, ResNet50, MobileNetV2, and NasNetMobile on downsampled 32×32×3 dermoscopic images. Their EfficientNetV2S model, combined with server-side voting, achieved accuracies of 89.83% and 90.64% under IID and non-IID settings, respectively, demonstrating that lightweight CNNs can maintain competitive diagnostic performance with substantially lower computational overhead.

To address the scarcity of labeled clinical data, ref. [17] proposed FedPerl, a semi-supervised peer-learning framework that leverages both labeled and unlabeled clinical samples by generating pseudo-labels for local unlabeled data. These pseudo-labels allow unlabeled images to be incorporated into local training, effectively increasing the amount of usable training data without compromising patient privacy. By forming similarity-driven peer committees with peer anonymization, clients collaboratively improve model performance while preserving data confidentiality. Evaluated on approximately 71,000 images from five repositories, FedPerl consistently outperformed state-of-the-art semi-supervised methods, including FedMatch, demonstrating strong robustness under non-IID and data-scarce settings. Subsequently, ref. [18] introduced FL-FS, which combines hybrid feature selection with local CNN architectures to improve classification accuracy on the ISIC 2019 and HAM10000 datasets while reducing training time. However, the framework was evaluated only in simulated FL environments and remains limited to conventional CNN-based feature representations. To support heterogeneous clinical annotations, ref. [19] developed FedMix, a label-agnostic segmentation framework capable of training across clients with diverse annotation types, ranging from dense pixel-level masks to weak image-level labels. FedMix employs Mixup-based augmentation, consistency regularization, and annotation-quality-aware aggregation to improve segmentation performance across heterogeneous annotations. However, these additional training operations require multiple forward passes and extra loss computations, substantially increasing computational cost and memory consumption while remaining susceptible to severe class imbalance.

In an effort to achieve early detection, ref. [32] demonstrated a deep CNN-based FL configuration that achieved up to 98% accuracy across the ISIC and PH2 [33] diagnostic databases, highlighting the clinical potential of decentralized medical AI while pointing out that thorough generalizability testing [20] is still necessary for wider real-world adoption. Ref. [21] proposed PMM-FL, a personalized multi-modal FL framework specifically intended to address heterogeneous or entirely absent imaging modalities [21], to manage multi-modal records and structural data gaps. PMM-FL achieved an accuracy of 92.32% on a multi-modal ISIC (2018–2024) subset using a pipeline that included hierarchical aggregation, attention-driven fusion, and learnable data imputation. This pipeline reduced communication traffic by 32.9% [21] while maintaining robust classification performance even under conditions of 30% modality absence.

A Model-Heterogeneous Semi-Supervised Federated (HSSF) learning framework for medical image segmentation was created by [22] to address localized data shortages and lessen annotation responsibilities. To optimize the use of unlabeled client databases, HSSF combines regularity condensation and fusion methods with a self-evaluating pseudo-label generating module. HSSF consistently outperformed current heterogeneous FL techniques when benchmarked on skin and polyp lesion datasets [22]. In a similar vein, ref. [23] introduced SkinFLNet to address privacy concerns. Using an optimized FL-VGG16 backbone [23], five deep CNN configurations were benchmarked on the ISBI 2016 dataset to achieve a peak accuracy of 92.08%. The work in [24] coupled an asynchronous FL framework with CNNs to get overcome the bandwidth limitations common to synchronous updates. By limiting frequent updates to shallow feature-learning layers and using temporally weighted aggregation to speed up global convergence, their approach reduces network traffic. This method outperformed normal FL baselines in accuracy and required less communication iterations when applied to the ISIC 2019 archive [24]. A similar [25] framework successfully reduced overall training times without sacrificing the model’s underlying diagnostic precision by integrating conventional FedAvg with a DenseNet201 backbone spread over four different clients [25]. DenseNet201 and VGG16 are commonly used as CNN backbones because their pretrained architectures provide strong feature extraction, stable optimization, and high classification performance on medical images. Recent advances have also explored image enhancement techniques to improve the quality of dermoscopic images before automated diagnosis. For example, ref. [34] proposed SAVE (Spectrum-Aided Visual Enhancement), which enhances diagnostically relevant visual characteristics of skin lesion images to improve the performance of deep learning-based skin cancer detection. Their study demonstrates that enhancing image quality can significantly benefit downstream classification models. While such approaches focus on improving the input image representation, the proposed FedSwin-LHTP framework addresses a complementary challenge by improving the computational efficiency and privacy preservation of transformer-based skin lesion classification in federated learning environments.

### 2.2. Vision Transformers and Advanced Visual Optimization

In recent developments in medical computer vision, dynamic token selection algorithms and architectural enhancements have been thoroughly studied. For instance, ref. [26] introduced Med-VCD (visual contrastive decoding) to sparsify token representations in medical Large Vision–Language Models (LVLMs). This reduces text-distorted perceptions and improves factual consistency across cross-modal tasks [26]. Similar to this, MedViTv2 balanced local and global feature extraction within transformer blocks by combining dilated attention layers with Kolmogorov–Arnold Network (KAN) blocks. This produced strong classification capabilities even when processing corrupted medical images [27]. Ref. [28] proposed FedViTBloc, which incorporates FL, ViTs, Fully Homomorphic Encryption (FHE), differential privacy, and blockchain protocols, extending these transformer concepts to secure environments [28]. The extra cryptography and blockchain layers provide significant computational and scalability issues, notwithstanding its efficacy on the HAM10000 dataset [28].

The CFWL model presented by [29] integrates the VGG16 backbone with traditional CNN features in a federated learning framework [29] when examining hybrid and federated techniques for skin cancer classification. The model achieved 90% accuracy on the ISIC dataset under non-IID settings, outperforming typical FedAvg and FedProx techniques, despite the fact that client failures and communication overhead continue to be issues at scale [29]. Similarly, ref. [35] demonstrated the efficacy of transformer architectures [30] by comparing pre-trained CNNs and ViTs for multi-class skin cancer classification. The ViT-based model achieved a maximum accuracy of 92.14%. Lastly, ref. [36] demonstrated the advantages of large-scale federated learning environments [31] by presenting a CNN-based classifier improved through local data augmentation and evaluated across up to 2000 simulated federated clients, increasing classification accuracy from 82.42% to 93.25%.

Although token pruning and sparsification techniques have been extensively studied to maximize model efficiencies [26,27], current paradigms do not work well in decentralized environments. For instance, a lightweight, MLP-based prediction network is used in the DynamicViT framework proposed by [37] to assess token importance and dynamically eliminate redundant visual patches during inference [37]. While highly efficient in centralized pipelines, DynamicViT is fundamentally incompatible with federated learning; its requirement for a homogeneous, centralized training set to optimize the prediction network violates core FL privacy mandates and leads to static pruning policies that fail to adapt to non-IID data variations [37].

### 2.3. Contributions

The primary contributions of this work are structured as follows:

- An optimized privacy-preserving federated architecture is developed to integrate the global representation capabilities of hierarchical Swin Transformers into decentralized clinical environments without requiring high-performance local server infrastructure.
- Lightweight Hessian-Inspired Token Pruning mechanism is proposed to dynamically prune less informative tokens using a hybrid first- and second-order saliency criterion, thereby reducing the computational and memory overhead of local transformer attention while maintaining classification performance.
- Comprehensive benchmarking on the multi-class HAM10000 dataset demonstrates that the proposed framework achieves strong diagnostic performance (exceeding 94% accuracy) while substantially reducing the computational and memory requirements of resource-constrained healthcare edge devices.

## 3. Materials & Methods

**Figure 1:**
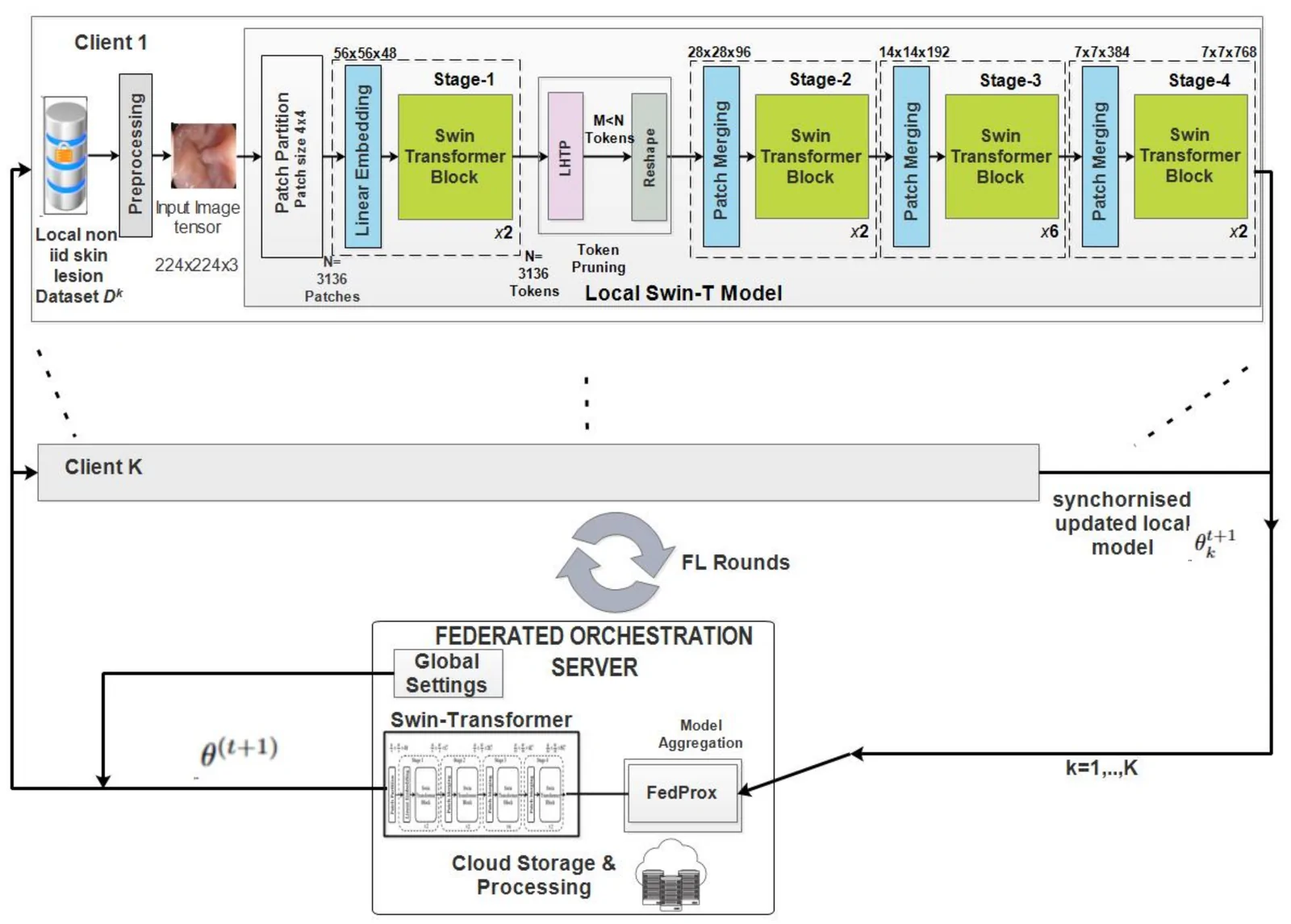
Proposed pipeline of FedSwin-LHTP framework.

Figure 1 illustrates the end-to-end pipeline of the proposed FedSwin-LHTP framework, comprising a central orchestration server and *K* clients that emulate a real-world multi-institutional environment. A Swin Transformer is adopted as the global backbone model because its hierarchical architecture and shifted window-based self-attention effectively capture both local and global image representations while maintaining lower computational complexity than conventional ViTs, making it well suited for FL. The federated training process is executed over multiple communication rounds. In each round, the central server distributes the global Swin Transformer model parameters, $\theta^{t}$, to a selected subset of *K* clients for local training. Each client then

1. Preprocesses the images of its local dataset $D^{k}$.
2. Passes the pre-processed images through its Swin Transformer backbone.
3. Applies the LHTP after Stage-1 of the local Swin-T model.
4. Uses only the pruned token embeddings (reshaped to a 28×28×96) grid for the remaining local training through Stages 2–4 of the Swin Transformer, which include patch-merging operations and shifted window attention blocks, followed by global average pooling and a linear classifier to produce skin lesion class predictions.
5. Transmits only the updated model weights (gradients) back to the central aggregation server, never sharing raw dermoscopic images, thus preserving patient privacy across participating institutions.

The central server then aggregates the weights from each client using Fed-Prox. This process continues for a given number of FL iterations, progressively improving the global skin lesion classification model while maintaining computational efficiency. The main components of the proposed framework are discussed in the following sub-sections.

### 3.1. Datasets

This study utilizes two public benchmark dermatological image datasets, i.e., HAM10000 [38] and ISIC2019 [39].

The HAM10000 (Human Against Machine with 10,000 training images) dataset is sourced by the Department of Dermatology, Medical University of Vienna. It is composed of 10,015 dermoscopic images categorized into seven skin lesion classes: AKIEC, BCC, BKL, DF, MEL, NV, and VASC. The raw images in this dataset are in RGB format with a consistent resolution of 600×450 pixels.

The ISIC2019 is a large-scale benchmark dermoscopic image dataset released as part of the International Skin Imaging Collaboration (ISIC) 2019 challenge for automated skin lesion classification. It is composed of 25,331 RGB images belonging to eight diagnostic categories, i.e., MEL, NV, BCC, actinic keratosis (AK), BKL, DF, VASC, and squamous cell carcinoma (SCC). The images in this dataset have varying resolutions ranging from 540×576 pixels up to 4499×6748 pixels.

**Figure 2:**
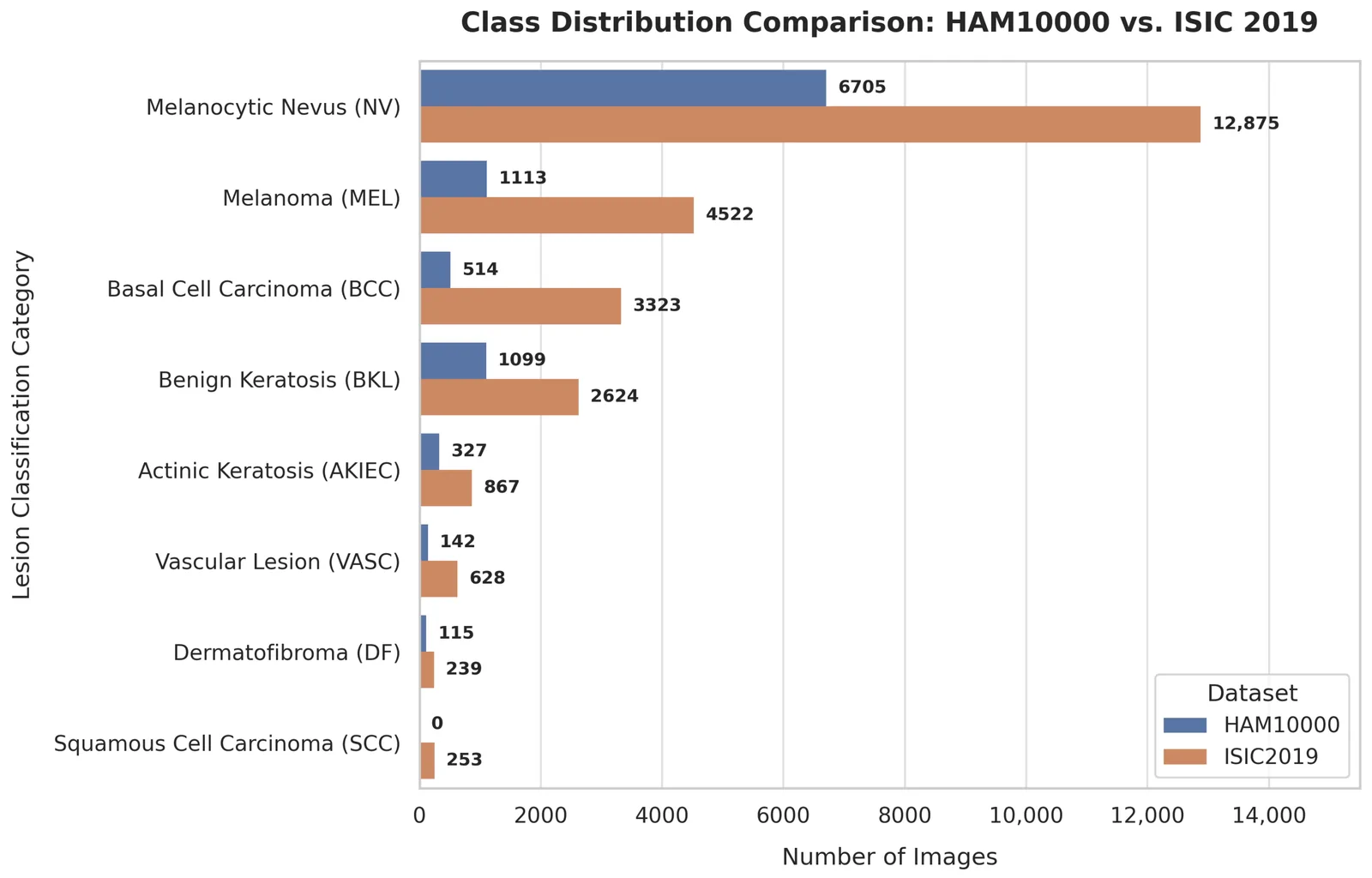
Class-wise distribution of images of HAM10000 and ISIC2019 Dataset.

Figure 2 demonstrates the class-wise distribution of images for both datasets. Both datasets show significant class imbalance, which biases the model toward majority classes, causing it to underperform on minority lesion categories. As a result, the model exhibits lower recall and higher misclassification rates for rare but clinically important lesions, ultimately reducing overall generalization and diagnostic reliability. In the HAM10000 dataset, the NV class dominates with 6705 images, accounting for approximately 67% of the total 10,015 samples, while rare classes such as DF contain only 115 images (1.1%), and VASC comprise 142 images (1.4%). The ISIC2019 dataset, exhibits comparable imbalance patterns where NV constitutes 12,875 images (41.2%), followed by MEL with 4522 images (21.3%), while rare categories such as DF contain only 239 images (1.1%) and VASC include 253 images (1.2%).

**Figure 3:**
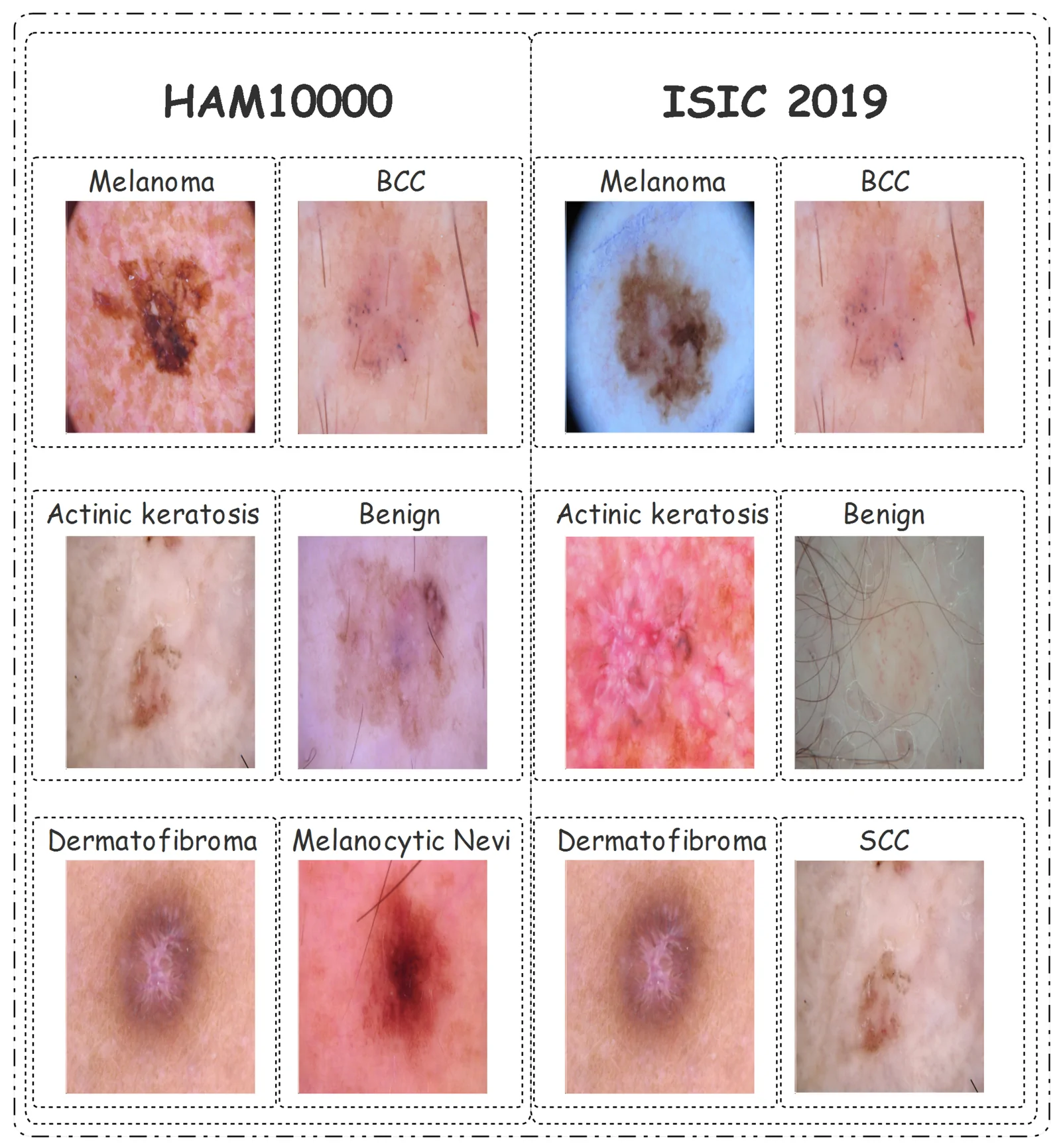
Selected samples of HAM10000 and ISIC2019 dataset (10× magnification).

Figure 3 presents representative samples from the HAM10000 and ISIC2019 datasets. Images from both datasets exhibit substantial visual and structural variability, posing significant challenges for both clinical diagnosis and automated deep learning systems. The distinction between benign and malignant lesions is often subtle, with several categories sharing highly similar visual characteristics. For instance, identical asymmetry, varying pigmentation patterns, and uneven borders might make it challenging to differentiate between benign NV and early-stage MEL. In a similar vein, benign BKL and BCC can resemble one another quite a deal, particularly in cases with little pigmentation. Furthermore, each diagnostic group exhibits a considerable degree of intra-class variability. For example, depending on the individual characteristics, anatomical location, and progression of the disease, a melanoma lesion may manifest as a large, multicolored, ulcerated lesion in one patient and as a small, uniformly pigmented macule in another. Intra-class variability and inter-class similarities further complicate automated skin lesion classification. While inter-class similarity results in overlapping feature representations across several lesion categories, decreasing the model’s discriminative power and raising the risk of misclassification, intra-class variability adds significant variance within the same lesion class.

### 3.2. Client-Side Pre-Processing

Since skin lesion data collected from different clinical institutions exhibit a non-independent and identically distributed (non-IID) nature, each client possesses a unique local data distribution with varying class proportions and imaging characteristics. Therefore, preprocessing is performed locally using a three-step pipeline comprising resolution normalization, data augmentation, and intensity standardization to ensure compatibility with the Swin Transformer architecture. Performing preprocessing at each client standardizes the input images while ensuring that raw patient data remain on the local device, thereby preserving patient privacy and adhering to the Federated Learning paradigm.

**Resolution Normalization:** Since the HAM10000 (600 × 450) and ISIC2019 (540 × 576 to 4499 × 6748) datasets contain images with heterogeneous resolutions, all images were resized to a fixed resolution of 224 × 224 pixels using bilinear interpolation. This resolution matches the input requirement of the pretrained Swin Transformer, ensuring consistent patch partitioning (4 × 4 patches producing 56 × 56 tokens), architectural compatibility, and efficient batch processing across all clients.

**Data Augmentation:** Spatial transformations (random rotations, flips), color adjustments (brightness ±20%, contrast ±15%, saturation ±10%), Gaussian blur (σ≤1.0), and mild elastic deformations were used to simulate real-world clinical variations in dermoscopic imaging in order to avoid overfitting and improve robustness.

**Intensity Standardization:** Pixel values were first scaled to the range [0,1] and then normalized using the ImageNet mean [0.485,0.456,0.406] and standard deviation [0.229,0.224,0.225]. ImageNet statistics were adopted because the Swin Transformer was initialized with ImageNet-pretrained weights. Using the same normalization aligns the input distribution with that used during pretraining, leading to faster convergence, improved training stability, and more effective transfer learning.

### 3.3. Swin Transformer Backbone

The proposed framework utilizes the Swin (Shifted Window) Transformer as a backbone architecture of feature extraction. The classical ViTs break the image into fixed-sized patches and compute global self-attention across all patches. This results in a quadratic complexity $O(N^{2})$ with respect to the number of patches *N*. In contrast, the Swin Transformer employs a hierarchical design employing window-based self-attention computation. This results in linear complexity in computational complexity O(N) with respect to the number of image tokens. As demonstrated in fig:pipeline1, the Swin transformer builds hierarchical feature representations through four stages, each progressively reducing spatial resolution while increasing channel depth. Given an input dermascopic image $I\in R^{224\times 224\times 3}$, the Swin Transformer computation is summarized as follows.

#### 3.3.1. Patch Partitioning & Linear Embedding

In the first step, the input image tensor is divided into non-overlapping patches of 4×4 pixels. This yields a tensor of size $\frac{224}{4}\times \frac{224}{4}=56\times 56$, with a total of 56×56=3136 patches. Each patch is then flattened into a vector of size 4×4×3=48 pixels. Each patch vector is then projected into a feature space with dimensions C=96, using a trainable linear embedding layer. This produces an embedded feature map $X\in R^{56\times 56\times 96}$.

#### 3.3.2. Stage 1

This stage is composed of two consecutive Swin Transformer blocks. The first block employs Window-based Multi-head Self-Attention (W-MSA), while the second block employs Shifted Window-based Multi-head Self-Attention (SW-MSA).

**Figure 4:**
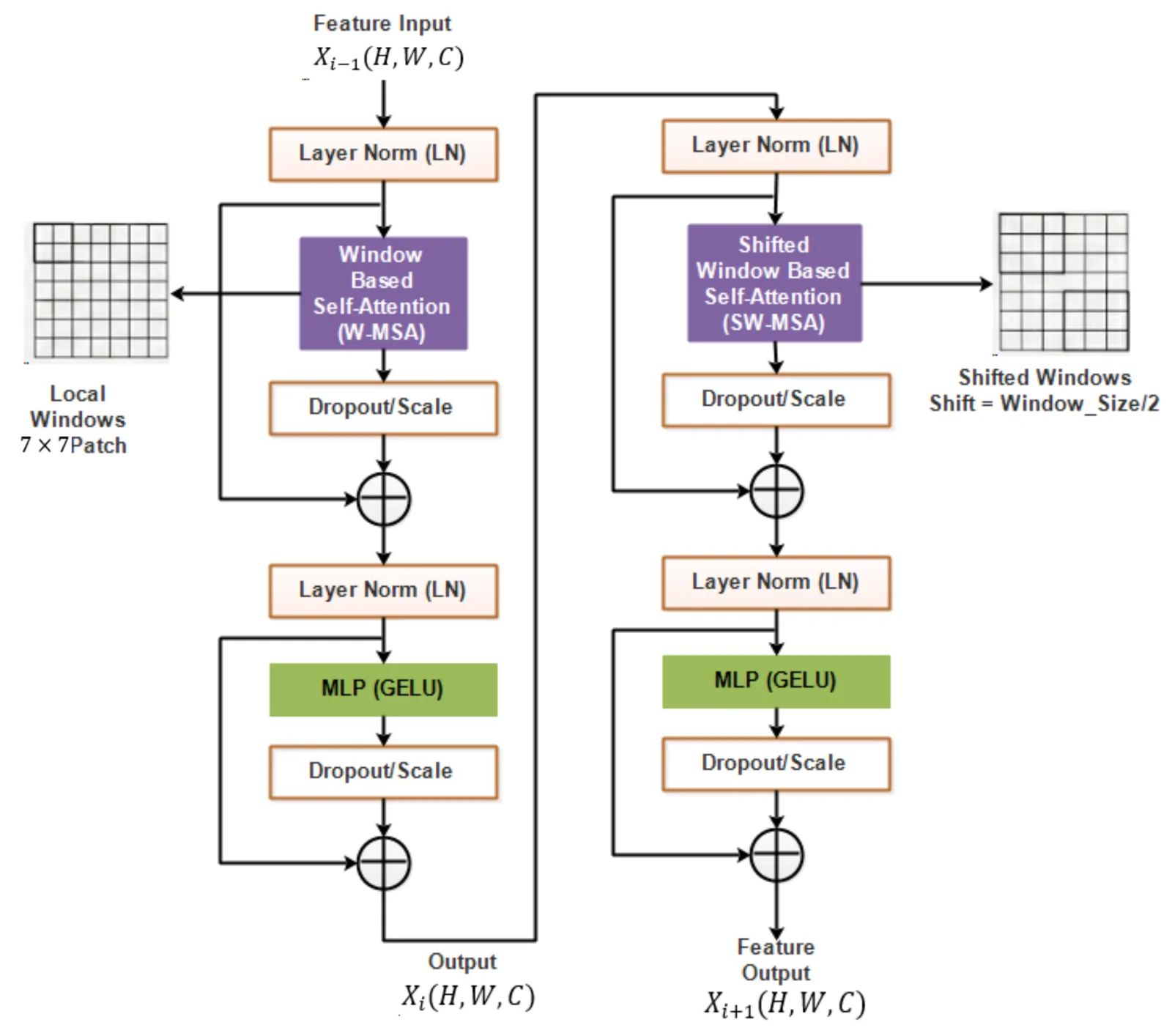
Consecutive Swin Transformer Blocks.

As demonstrated in Figure 4, each Swin-T block is composed of Layer Norm (LN), followed by the respective attention mechanism (W-MSA or SW-MSA), a residual connection, another Layer Norm, a two-layer Multi-Layer Perceptron (MLP) with GELU activation and expansion factor of 4, and a final residual connection. Mathematically, the dataflow through these blocks is computed as:

Block l

(1) $$\begin{array}{ccc} {\hat{X}}^{l} & = & W-\mathrm{MSA}\left(\mathrm{LN}\left(X^{l-1}\right)\right)+X^{l-1} \end{array}$$

(2) $$\begin{array}{ccc} X^{l} & = & \mathrm{MLP}\left(\mathrm{LN}\left({\hat{X}}^{l}\right)\right)+{\hat{X}}^{l} \end{array}$$

Block l + 1

(3) $$\begin{array}{ccc} {\hat{X}}^{l+1} & = & \mathrm{SW}-\mathrm{MSA}\left(\mathrm{LN}\left(X^{l}\right)\right)+X^{l} \end{array}$$

(4) $$\begin{array}{ccc} X^{l+1} & = & \mathrm{MLP}\left(\mathrm{LN}\left({\hat{X}}^{l+1}\right)\right)+{\hat{X}}^{l+1} \end{array}$$

The W-MSA partitions the 56×56 feature map into non-overlapping windows of size 7×7, resulting in (56/7)×(56/7)=8×8=64 number of windows, each containing 7×7=49 tokens. Self-attention is calculated within each window independently of one another. The subsequent SW-MSA shifts the window partitioning by half the window size (3 pixels), enabling cross-window connections and allowing information flow between neighboring windows. The output of Stage 1 remains $X_{1}\in R^{56\times 56\times 96}$, preserving the original token count of N=3136.

#### 3.3.3. Stage 2

Stage 2 precedes with a patch-merging layer, which decreases the spatial resolution while increasing the channel dimension, producing a feature map of size (28×28×192). This stage also employs two Swin transformer blocks consisting of W-MSA and SW-MSA mechanisms. At this stage, the network is able to capture intermediate-level features of the skin lesion, including pigmentation distributions, border irregularities, and local structural patterns. The output tensor of this stage has dimensions (28×28×192).

#### 3.3.4. Stage 3

In this stage, the patch-merging layer transforms the input tensor into (14×14×384). This stage constitutes the deepest component of the Swin-T containing six alternating W-MSA and SW-MSA blocks. The increase in channel dimensionality complemented by a large receptive field enables the network to understand complex lesion morphological features and long-range dependencies. Consequently, diagnostically significant characteristics such as lesion asymmetry, heterogeneous pigmentation, structural disorder, and texture variations can be effectively represented. The output tensor of Stage 3 has dimensions remains (14×14×384).

#### 3.3.5. Stage 4

As stated earlier, the third patch-merging layer reduces the spatial dimensions of feature map to 7×7 while increasing the channel dimension to 768. This stage is composed of two Swin-T blocks employing W-MSA and SW-MSA operations. At this stage, the network learns highly abstract and rich semantic features that encode global contextual relationships required to distinguish visually similar lesion categories, such as MEL and NV or BCC and BKL. The output feature map retains dimensions (7×7×768).

Finally, the last feature map is processed by global average pooling to generate a compact 768-dimensional feature vector. This representation is then passed to a fully connected classification layer and softmax activation function to generate the ultimate probability distribution over the skin lesion categories.

#### 3.3.6. Window-Based Self-Attention Mechanism

The Swin-T employs a window-based self-attention mechanism to efficiently model spatial relationships among image patches. Unlike conventional ViTs, which compute global self-attention across all tokens, Swin Transformer restricts attention computation to non-overlapping local windows, substantially reducing computational complexity while preserving important contextual information.

For an input feature representation X, the query (Q), key (K), and value (V) matrices are generated through learnable linear projections:

(5) $$Q=XW_{Q},\;K=XW_{K},\;V=XW_{V}$$

The attention operation within each window is computed as:

(6) $$\mathrm{Attention}\left(Q,K,V\right)=\mathrm{Softmax}\left(\frac{QK^{T}}{\sqrt{d_{k}}}+B\right)V$$

where $d_{k}$ denotes the key dimension and B represents the relative positional bias. Multiple attention heads operate in parallel to capture diverse lesion characteristics, including pigmentation patterns, texture variations, and border irregularities.

To facilitate information exchange across neighboring windows, Swin-T alternates W-MSA with SW-MSA. The shifted-window strategy progressively expands the receptive field and enables effective modeling of both local and global contextual information. By restricting attention computation to fixed-size windows, the Swin-T achieves near-linear computational complexity with respect to image size, making it well suited for high-resolution skin lesion analysis in federated learning environments.

### 3.4. LHTP—Lightweight Hessian-Inspired Token Pruning

Although the window-based self-attention mechanism of the Swin-T substantially reduces computational complexity, a considerable number of patch tokens remain redundant. In dermoscopic images, these redundant tokens predominantly correspond to healthy skin, background regions, illumination artifacts, and hair structures, with only a small subset of tokens representing diagnostically significant lesion areas. These uninformative tokens constitute enormous computational and memory complexity without significant impact on the model’s diagnostic performance. To address this issue, the proposed framework introduces an LHTP module which is inserted immediately after Stage 1 of the Swin Transformer to dynamically reduce the number of tokens from N=3136 to (K<N), while preserving diagnostically relevant information for skin lesion classification.

#### 3.4.1. Problem Formulation

Let $X\in R^{N\times C}$ represents the flattened token sequence after Stage 1 of the Swin Transformer, where N=3,136 and C=96. Let L(X) represent the classification loss computed at the output layer of the network for X. Given the predicted class probability vector $\hat{y}$ and the corresponding true label *y*, the loss is evaluated using the cross-entropy objective function:

(7) $$L\left(X\right)=-\frac{1}{N}\sum_{i=1}^{N}\sum_{c=1}^{C}w_{c}y_{c}\log\left({\hat{y}}_{c}\right)$$

where *C* denotes the total number of lesion classes, $y_{c}$ is the ground-truth label, ${\hat{y}}_{c}$ is the probability of the predicted class and $w_{c}$ is the weight associated with class c. $w_{c}$ is defined as:

(8) wc=1N∑j=1C1Nj.

The weighted cross-entropy loss function assigns greater penalties to errors made on under-represented lesion classes and smaller penalties to errors made on majority classes. This weighting addresses the serious class imbalance in the HAM10000 and ISIC2019 datasets and enhances the learning of more discriminative representations for the rare lesion categories. The gradient of the weighted loss with respect to the $i^{th}$ token representation is given by:

(9) $$\nabla L_{i}=\frac{\partial L_{WCE}\left(X\right)}{\partial x_{i}}$$

Consequently, the variation in L(X) resulting from the removal or perturbation of a token $x_{i}$ provides an estimate of that token’s contribution to the final classification decision and mathematically expressed using Taylor Expansion as:

(10) $$L\left(X-x_{i}\right)=L\left(X\right)-\nabla L_{i}^{⊤}x_{i}+\frac{1}{2}x_{i}^{⊤}H_{ii}x_{i}+O(∥x_{i}{∥}^{3})$$

$\nabla L_{i}\in R^{C}$ is the gradient of the loss function with respect to token *i*, and $H_{ii}\in R^{C\times C}$ is the Hessian block corresponding to token *i* and $O(∥x_{i}{∥}^{3})$ represents higher-order residual terms which can be safely neglected. Tokens that induce a larger increase in the loss are considered more informative, whereas tokens associated with negligible loss variation are regarded as redundant and can be safely pruned with minimal impact on diagnostic performance. In the above expression, the Hessian matrix H consists of second-order partial derivatives, mapping the local curvature of the loss surface. Subtracting L(X) from both sides isolates the actual change in the loss $\Delta {\left(L\right)}_{i}$:

(11) $$\Delta L_{i}=L\left(X-x_{i}\right)-L\left(X\right)\approx -\nabla L_{i}^{⊤}x_{i}+\frac{1}{2}x_{i}^{⊤}H_{ii}x_{i}$$

Following the common assumption adopted in second-order optimization and Fisher Information approximations, local models that have undergone several communication rounds are assumed to operate in a near-stationary regime where $E[\nabla L_{i}]\approx 0,\forall i$ [40]. This approximation becomes increasingly valid as local models approach stationary regions during the later communication rounds and is employed only as a computationally efficient surrogate for token saliency estimation. Consequently the first-order term $E[\nabla L_{i}^{⊤}x_{i}]\approx 0$, the loss change is then approximated by the Hessian term as:

(12) $$\Delta L_{i}\approx \frac{1}{2}x_{i}^{⊤}H_{ii}x_{i}$$

which is used to define the Hessian’s inspired token saliency score $S(x_{i})$ as:

(13) $$S\left(x_{i}\right)\approx \Delta L_{i}\approx \frac{1}{2}x_{i}^{⊤}H_{ii}x_{i}$$

Direct computation of the Hessian matrix H is computationally prohibitive for transformer architectures because of the large number of tokens and model parameters involved.

For Stage 1 of the Swin Transformer, the feature representation consists of 3136 tokens, each with a 96-dimensional embedding. Direct computation of the corresponding Hessian matrix would require evaluating second-order interactions among approximately $3.01\times 10^{5}$ variables, resulting in a Hessian containing nearly $9.06\times 10^{10}$ entries and requiring hundreds of gigabytes of memory. Furthermore, direct computation of the Hessian matrix is computationally prohibitive because both the number of second-order derivatives and the memory requirements scale quadratically with the number of model variables. These computational and memory requirements are particularly prohibitive in federated learning environments, where client devices typically operate under resource constraints.

#### 3.4.2. Hessian-Free Approximation

Instead of explicitly computing the Hessian matrix, the proposed framework adopts a commonly used second-order approximation based on the relationship between the Hessian H, Fisher Information Matrix (F), and gradient covariance. Near a local optimum, the Hessian can be approximated using the expected outer product of gradients as [41]:

(14) $$H\approx F=E[\nabla L\cdot \nabla L^{⊤}]$$

which provides a computationally efficient estimate of curvature information.

Accordingly, the diagonal Hessian block corresponding to the *i*-th token can be approximated as

(15) $$H_{ii}\approx E\left[\nabla L_{i}\nabla L_{i}^{⊤}\right]$$

where ∇i denotes the gradient associated with token *i*. Taking the trace of the diagonal block yields a scalar measure of local curvature:

(16) $$\mathrm{Tr}\left(H_{ii}\right)\approx E[∥\nabla L_{i}{∥}_{2}^{2}]-∥E\left[\nabla L_{i}\right]{∥}_{2}^{2}=\mathrm{Var}\left(\nabla L_{i}\right)$$

where $\mathrm{Var}(\nabla L_{i})$ is the variance of $\nabla L_{i}$ across its C=96 channels:

(17) $$\mathrm{Var}\left(\nabla L_{i}\right)=\frac{1}{C}\sum_{c=1}^{C}{\left(\frac{\partial L}{\partial X_{i,c}}-\mu_{i}\right)}^{2},\;\mu_{i}=\frac{1}{C}\sum_{c=1}^{C}\frac{\partial L}{\partial X_{i,c}}$$

#### 3.4.3. Hessian-Free Saliency Score

Substituting this approximation into the Hessian-Inspired saliency score and assuming $∥x_{i}∥=1$:

(18) $$S\left(x_{i}\right)\approx \frac{1}{2}\mathrm{Var}\left(\nabla L_{i}\right)$$

The above formulation captures the second-order importance of tokens, but relying on the curvature information may not be sufficient in early stages of training and in highly heterogeneous federated settings where token gradients can be significant. Thus, we add a first-order sensitivity term to the second-order criterion. The final token saliency score is defined as:

(19) $$S_{i}=\lambda_{1}\;\cdot {|\nabla L_{i}∥}_{2}+\lambda_{2}\cdot \mathrm{Var}\left(\nabla L_{i}\right)$$

In all experiments, $\lambda_{1}=1$ and $\lambda_{2}=0.5$, which were selected empirically based on validation performance. Also, $\lambda_{2}=0.5$ matches the $\frac{1}{2}$ coefficient from Taylor expansion. This formulation is completely Hessian-free, requiring only:

- Gradient norm $∥\nabla L_{i}{∥}_{2}$ (first-order information)
- Gradient variance $\mathrm{Var}(\nabla L_{i})$ (curvature proxy)

Both quantities are derived from the same gradients that are already computed during standard backpropagation, requiring no additional computational overhead.

#### 3.4.4. The LHTP Algorithm

**Figure 5:**
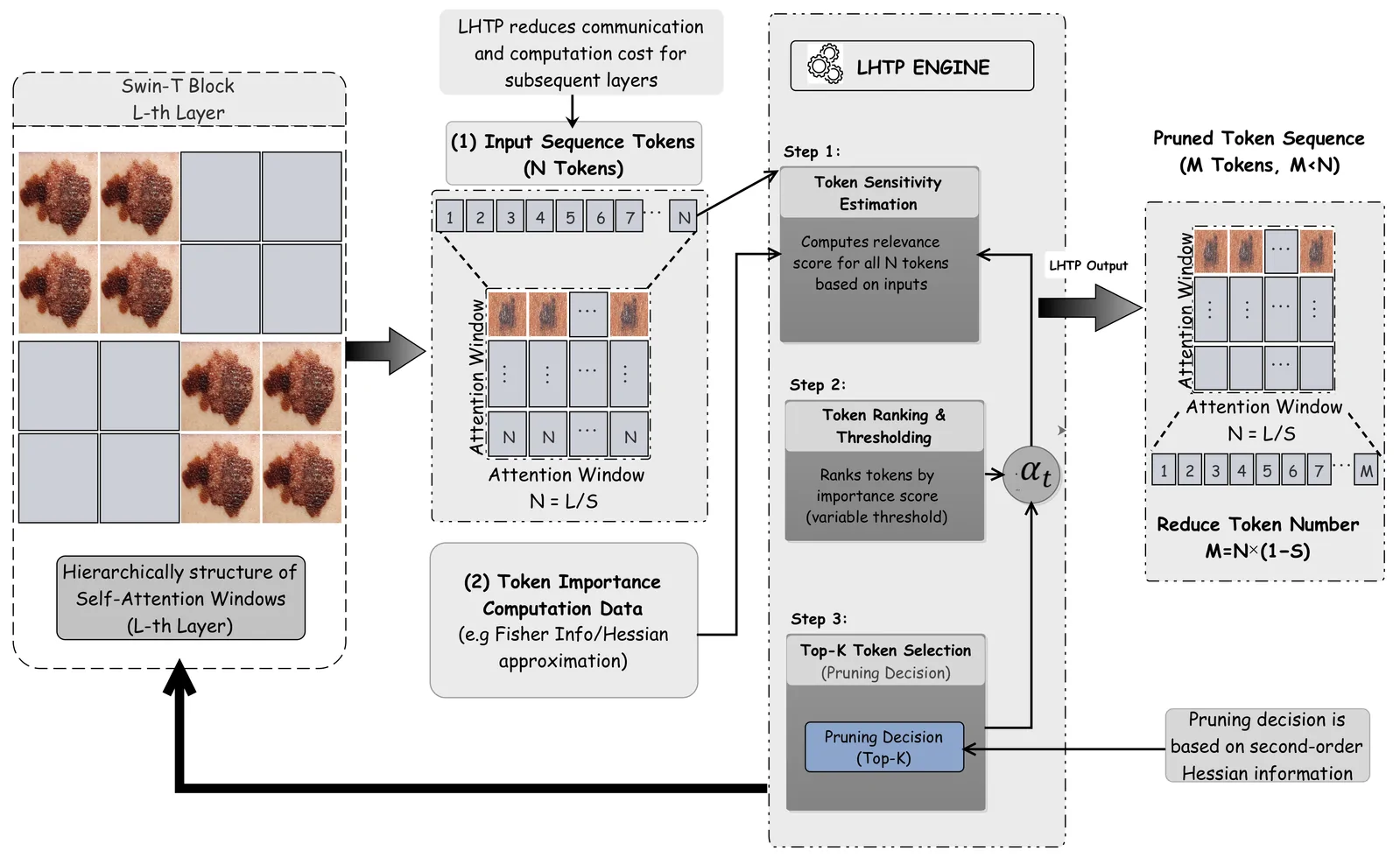
LHTP Algorithm Flow Diagram.

Figure 5 shows a generic flow diagram of LHTP algorithm. The proposed LHTP module is incorporated immediately after Stage 1 of the Swin Transformer to prune less informative tokens before they are processed by deeper transformer layers. The main steps of LHTP are summarized as follows.

Step 1: Stage-1 Token Generation

Given an input dermoscopic image (X), the Patch Embedding layer and Stage 1 of the Swin Transformer generate a contextualized token set

$$T=t_{1},t_{2},\ldots ,t_{N}$$

where N=3136 is the total number of Stage-1 tokens.

Step 2: Classification Loss Computation

Using Equation (7), a forward pass over the network is used to calculate the classification loss.

Step 3: Gradient Estimation

The classification loss is backpropagated to obtain the gradient associated with each token:

(20) $$\nabla L_{i}=\frac{\partial L}{\partial t_{i}}$$

where $\nabla L_{i}$ measures the sensitivity of the loss function to perturbations in token $t_{i}$.

Step 4: Hessian-Inspired Importance Estimation

The importance score of the $i^{\mathrm{th}}$ token is computed using (19)

Step 5: Token Ranking

The computed importance scores are used to rank all tokens in descending order:

(21) $$S_{\left(1\right)}\geq S_{\left(2\right)}\geq \ldots \geq S_{\left(N\right)}$$

where higher scores indicate more informative tokens.

Step 6: Adaptive Threshold Selection

Instead of using a predefined pruning ratio, the proposed LHTP framework determines the pruning threshold adaptively from the distribution of token importance scores within each input sample. Let $S_{i}$ denote the importance score of token $t_{i}$. The adaptive threshold δ is computed from the score distribution as

(22) $$\delta =\mu_{S}-\beta \sigma_{S}$$

where $\mu_{S}$ and $\sigma_{S}$ denote the mean and standard deviation of the token importance scores, respectively, and β is a sensitivity parameter controlling the aggressiveness of pruning. Tokens with importance scores below δ are considered less informative and become candidates for pruning.

The quantity of retained tokens can change based on lesion characteristics and picture complexity as a result to this adaptive method. As a result, although redundant representations are pruned more aggressively, informative pictures naturally retain more tokens, leading to a dynamic and data-dependent token reduction process.

Step 7: Dynamic Binary Mask Generation

Based on the adaptive threshold δ, a binary mask is generated for each token according to

(23) $$m_{i}=\left\{\begin{array}{cc} 1, & S_{i}\geq \delta \\ 0, & S_{i}<\delta \end{array}\right.$$

where $m_{i}=1$ indicates that token $t_{i}$ is retained, while $m_{i}=0$ denotes that the token is suppressed. Unlike conventional token-pruning approaches that rely on a predefined pruning ratio, the proposed LHTP framework determines token retention dynamically according to the distribution of token importance scores within each input sample.

The total number of retained tokens is therefore given by

(24) $$K=\sum_{i=1}^{N}m_{i},$$

where *N* denotes the total number of Stage-1 tokens. Since the value of *K* depends on the image content and the corresponding importance-score distribution, the effective pruning ratio is automatically determined as

(25) $$r=1-\frac{K}{N}.$$

As a result, samples with more redundant information experience more extensive token suppression, while images with richer and more discriminative lesion features typically retain more informative tokens. The proposed framework may allocate computational resources in accordance with the complexity of each input image while maintaining characteristics that seem relevant for effective skin-lesion classification thanks to this adaptive pruning technique. In practice, the adaptive thresholding mechanism retains approximately 40% of Stage-1 tokens on average, corresponding to an effective pruning ratio of approximately 60%. The exact number of retained tokens is determined dynamically based on the distribution of token-importance scores and the underlying complexity of the lesion representation.

Step 8: Window Constraint for Structure-Aware Pruning

To preserve the local attention structure of the Swin Transformer, each attention window $W_{j}$ is constrained to retain a minimum number of active tokens:

$$\sum_{i\in W_{j}}m_{i}\geq \tau$$

where τ denotes the minimum token retention threshold.

Step 9: Token Masking

The generated mask is applied to the Stage-1 token representations:

(26) $${\tilde{t}}_{i}=m_{i}⊙t_{i}$$

where ⊙ denotes element-wise multiplication.

Step 10: Forward Propagation

The masked token set

(27) $$\tilde{T}={\tilde{t}}_{1},{\tilde{t}}_{2},\ldots ,{\tilde{t}}_{N}$$

is forwarded to Stages 2–4 of the Swin Transformer for subsequent feature extraction and classification. By suppressing low-importance tokens while preserving window-level contextual information, the proposed LHTP module reduces computational overhead and memory consumption with minimal impact on diagnostic performance.

### 3.5. Federated Aggregation Using FedProx

Due to heterogeneous client data distributions in FL, the local models may exhibit high divergence from the global modal. Therefore, the conventional FedAvg may result in degraded convergence behavior. The proposed FL framework uses the FedProx algorithm to stabilize model convergence. In a given FL communication round *t*, the central orchestration server broadcasts its global Swin transformer model $\theta^{t}$. Each client *k* then performs local training on its local dataset $D^{k}$. However, unlike conventional FedAvg, the FedProx introduces additional proximal regularization term into the client local objective function;

(28) $$F_{k}\left(w\right)=\frac{1}{|D_{k}|}\sum_{X\in D_{k}}L_{WCE}\left(X;\theta_{k}^{t}\right)+\frac{\mu }{2}{∥\theta_{k}^{t}-\theta^{t}∥}_{2}^{2}$$

where $L(X;\theta_{k}^{t})$ is the classification loss of client k’s model, i.e., $\theta_{k}$ for image sample $X\in D_{k}$. In the above formulation, the first term minimizes the average classification loss over the local dataset, while the second term is a proximal regularization term that penalizes excessive deviation from the current global model $\theta^{t}$. The proximal coefficient μ controls the strength of this regularization and helps reduce client drift under heterogeneous non-IID data distributions.

Since the proposed LHTP module relies on gradient-based token importance estimation, excessive client drift may lead to unstable token-selection behavior across institutions. By constraining local model updates through FedProx, the resulting gradient distributions become more stable, thereby improving the reliability and consistency of Hessian-inspired token importance estimation under heterogeneous federated settings.

## 4. Results

### 4.1. Experimental Setup

The simulation framework shown in fig:pipeline1 comprises a central aggregation server and multiple clients representing distributed healthcare institutions. During each round of federated learning, the server shares the global Swin-T model with all participating clients so they can train it on their own private datasets. In Stage 1, clients filter out redundant tokens utilizing their specific data using an LHTP module. This step really helps reduce the computational costs while keeping the raw patient data securely on-site. To cope with the fact that every client has vastly diverse data sets, we rely on the FedProx objective during local training. This in effect anchors the local updates to the global model, preventing the individual models from diverging too much and improving overall convergence. Clients only send back their updated parameters after local training, never sensitive patient images. The server then aggregates these updates into a new global model and communicates it back to the clients for the next round. Such a setup enables genuine collaborative learning avoiding the sensitive data to be in one place. Finally, to ensure that our experimental results are robust and statistically sound, we run all the federated training simulations five times (N=5) with different random seeds. Results are presented as the mean ± standard deviation.

All experiments were conducted in a privacy-preserving federated environment. Raw patient images were retained strictly on local client devices, with only model parameters transmitted to the central server for aggregation. Each client node featured an Intel Core i7-12700K processor (12 cores, 3.6 GHz; Intel Corporation, Santa Clara, CA, USA), 32 GB DDR4 RAM, a 1 TB NVMe SSD, and an NVIDIA RTX 3080 GPU (10 GB VRAM; NVIDIA Corporation, Santa Clara, CA, USA) running Ubuntu 22.04 LTS. GPU acceleration was critical for training the computationally intensive Swin Transformer architecture, particularly to handle its self-attention mechanisms and large-scale matrix computations.

The central aggregation server utilized comparable computational resources, supplemented with enhanced storage and high-bandwidth networking capabilities to minimize communication overhead. Federated orchestration was driven by the Flower framework, which seamlessly managed client participation, communication-round scheduling, parameter synchronization, and global model aggregation.

### 4.2. Dataset Distribution

The HAM10000 and ISIC2019 datasets were further divided among clients using a Dirichlet distribution [42] to simulate realistic federated learning environments. We have purposely created this scenario to simulate the non-IID nature of actual medical data, with each client having different class distributions, which is a realistic representation of the diverse patient populations across different hospitals. In all experiments, we fixed the concentration parameter α to 0.5 to capture a moderate level of this statistical heterogeneity. For unbiased evaluation, each dataset was randomly divided into 80% training set and 20% testing set. The training part was shared among the clients while the centralized test set was used to evaluate the performance of the global model. As detailed in Table 2, experiments were conducted using configurations of 5 and 10 clients. These settings allow us to evaluate the proposed framework at different federation scales—representing a collaboration among a small group of hospitals and a larger, more distributed healthcare network, respectively.

The Swin Transformer backbone is trained and fine-tuned for skin lesion datasets using transfer learning, with main hyperparameters listed in Table 3. These parameters are meticulously selected to achieve a balance between convergence performance and computational efficiency in the federated environment. A mini-batch size of 8 and three local training epochs were adopted to reduce overfitting while maintaining efficient client-side optimization. The learning rate was set to ($1\times 10^{-5}$), and local model updates were optimized using SGD with momentum. Training was conducted for 50 communication rounds, during which participating clients performed local optimization using the FedProx objective function. After each communication round, the updated client models were transmitted to the central server, where they were aggregated to construct an updated global model that was subsequently redistributed for the next round of federated training.

### 4.3. Results of HAM10000 Dataset

#### 4.3.1. Scenario 1: K=5,α=0.5

**Figure 6:**
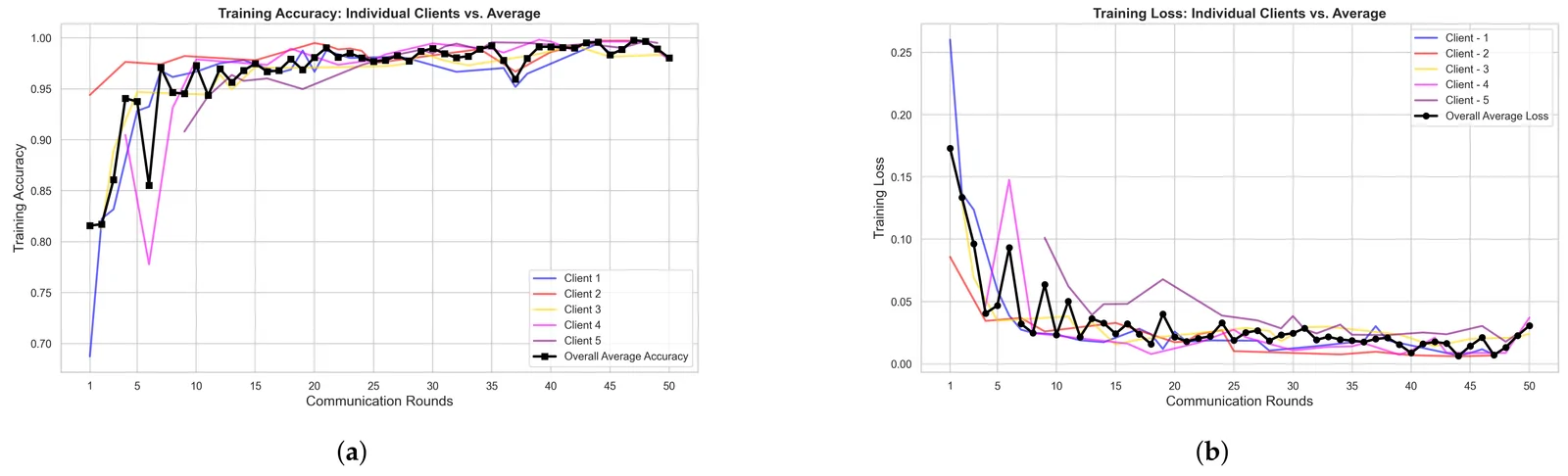
Local training accuracy and Loss plots for K=5 clients across federated learning rounds for the HAM10000 dataset with α=0.5. (**a**) Training Accuracy; (**b**) Training Loss.

To evaluate the convergence characteristics and robustness of the proposed FedSwin-LHTP framework, training performance was monitored over 50 federated communication rounds using the HAM10000 dataset under a heterogeneous non-IID setting (K=5, α=0.5). The evolution of client-wise and average training accuracies is illustrated in Figure 6a. During the initial communication rounds, noticeable variability is observed among the participating clients, with training accuracies ranging approximately from 68% to 95%. Such variations are expected under Dirichlet-based data partitioning, where clients possess different local class distributions and lesion characteristics. However, as training progresses, the gap between clients performance reduces gradually. This indicates better alignment of local model updates. The average training accuracy is above 95% after approximately 15 communication rounds and continues to grow steadily, finally stabilizing in the 98–99% range during the last steps of training. The close proximity of the single client accuracies to the global average indicates the effectiveness of the selected federated optimization strategy in reducing client divergence and promoting the learning of a consistent global representation despite data heterogeneity.

Figure 6b presents the training loss curves for the above experiments, which further validates the convergence behavior observed. The average training loss drops quickly in the first communication rounds from around 0.17 to less than 0.05 in the first few rounds. The loss then gradually decreases at a slower pace before stabilizing at around 0.01 to 0.03 at the end of training. We notice some moderate variations between individual clients during the intermediate stages, but these tend to disappear as the training progresses. The optimization appears more stable as the variance between clients decreases when the individual clients’ loss trajectories are more similar to the average loss trajectory. The general trends of accuracy and loss indicate the proposed framework can converge stably under the heterogeneous federated settings without persistent instability or divergence.

The observed training behavior also provides details about the effect of the LHTP mechanism in the federated Swin Transformer architecture. From the relatively smooth convergence of the accuracy and loss curves, it seems that the pruning process does not induce much optimization instability during training. This suggests that the Hessian-guided token selection strategy can generally identify and remove less influential spatial tokens while preserving tokens that meaningfully contribute to lesion representation. In this way, this reduction of token redundancy is obtained without disturbing the overall learning dynamics of the model. The training curves themselves do not directly measure the quality of retained representations, but suggest that the pruning decisions are still broadly compatible with the optimization objective across participating clients. The normalized aggregated confusion matrix of the converged model is shown in Figure 8a for further analysis of the classification performance. In general the matrix is significantly diagonally dominant. This implies that the framework is able to maintain reliable discrimination over the seven lesion categories despite the difficulties of non-IID data distribution. The class-wise recall values range from 89.69% to 97.41%, indicating that most of the lesion samples are correctly identified. Among the evaluated categories, NV (97.41%), VASC (95.92%), MEL (94.95%) and BKL (94.01%) achieve relatively higher recall values, while DF (89.69%) and BCC (90.76%) show relatively lower recognition rates. The main misclassifications are between DF and MEL (4.12%), between DF and AKIEC/BKL (3.09%) and between BCC and DF (3.55%) and NV (3.08%). Additionally, there is a moderate confounding between AKIEC and BKL (3.37%) and BCC (3.00%). These error patterns are consistent with the visual similarities that can exist between some categories of skin lesions, and have been frequently reported in skin lesion classification studies. Importantly, the MEL class achieves a recall of 94.95% with relatively limited confusion across the remaining categories, meaning that most melanoma samples are successfully recognized.

Overall, the findings shown in Figure 6a,b and Figure 8a indicate that the suggested FedSwin-LHTP framework can maintain strong classification performance under heterogeneous data distributions while achieving stable federated convergence. The Hessian-Inspired token pruning mechanism can reduce token redundancy without significantly compromising the discriminative information needed for skin-lesion classification, as evidenced by the combination of consistent optimization behavior, decreased inter-client variability, and high class-wise recognition rates.

#### 4.3.2. Scenario 2: K=10,α=0.5

To further evaluate the scalability of the proposed FedSwin-LHTP framework, experiments were conducted using K=10 decentralized clients under the same non-IID data partitioning setting (α=0.5). Increasing the number of participating clients while maintaining a fixed dataset size introduces a more challenging federated learning environment, as the available training data become more fragmented and local class distributions tend to be increasingly imbalanced. Such situations may negatively impact optimization stability and model generalization, and they often increase statistical heterogeneity across customers. However, during the federated learning process, the suggested framework exhibits consistent training behavior and sustains robust classification performance.

**Figure 7:**
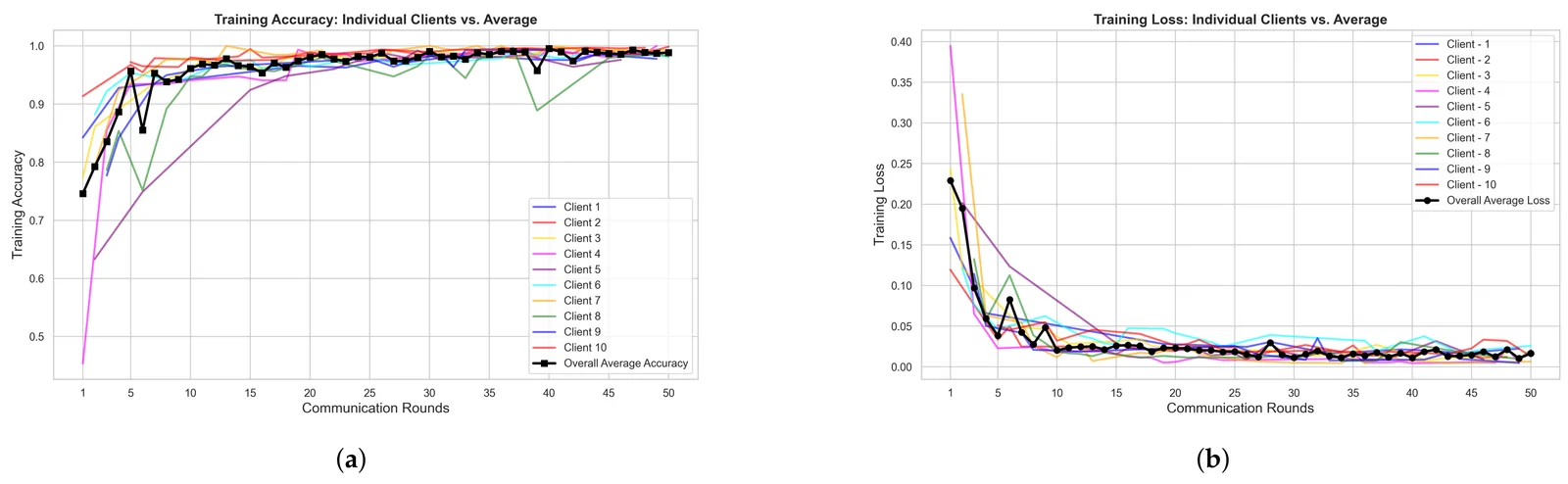
Local training accuracy and Loss plots for K=10 clients across federated learning rounds for the HAM10000 dataset with α=0.5. (**a**) Training Accuracy; (**b**) Training Loss.

Figure 7a,b show the framework’s convergence characteristics. The increased heterogeneity associated with tighter data partitioning is reflected in the higher variability among client models during the early communication rounds as compared to the K=5 setup. For example, Client 7 records a loss value close to 0.34 during the second communication round, but Client 4 shows a rather high training loss of around 0.40 and a training accuracy near 45% during the first round. These variations are consistent with the expected behavior of federated systems working in non-IID conditions, where the local optimization trajectories are influenced by the data distributions unique to each client. Despite these initial discrepancies, as training progresses, the client models tend to converge, with the accuracy and loss curves getting closer. As demonstrated by the absence of persistent oscillations or divergence during the course of the 50 communication rounds, the optimization process is also stable in the more challenging decentralized setting. This behavior indicates that the negative effect of increasing client heterogeneity can be mitigated by combining the Swin Transformer backbone, the LHTP mechanism, and the selected federated optimization method.

**Figure 8:**
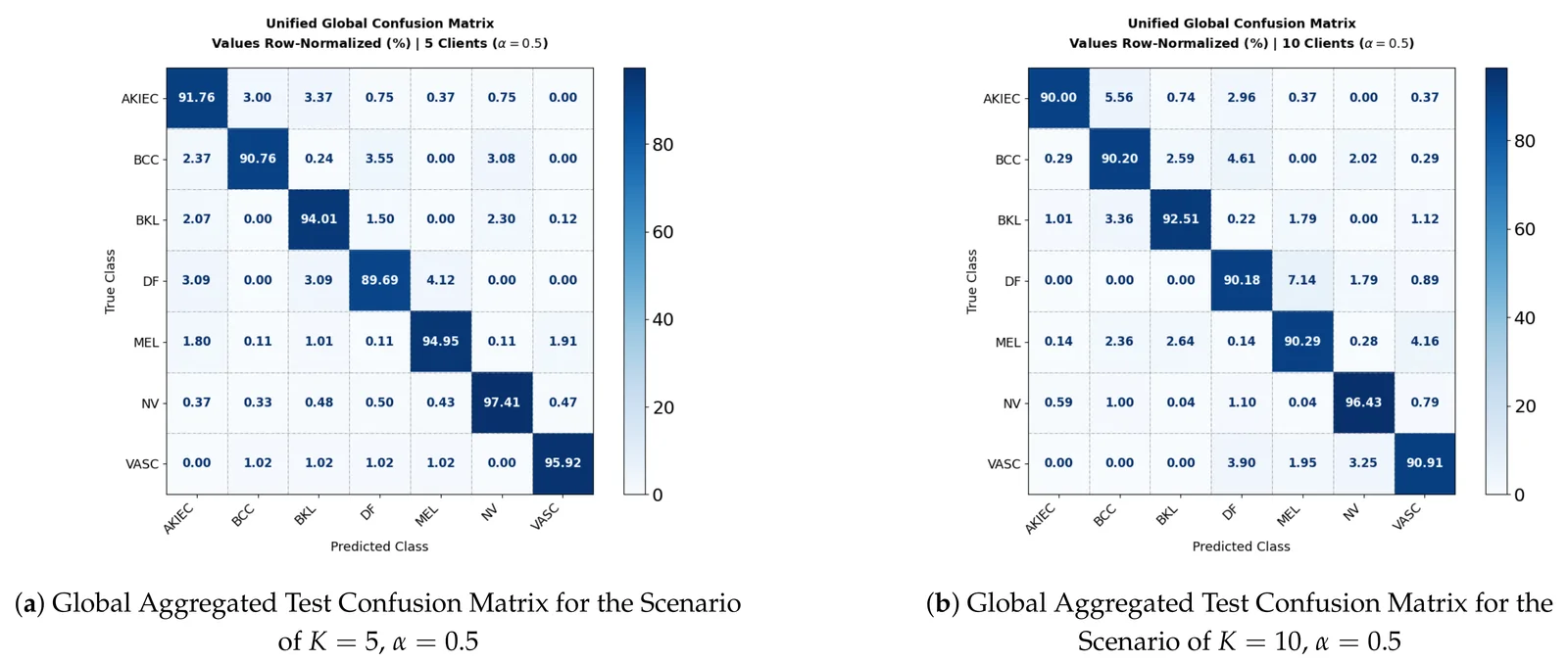
Global confusion matrices of the federated model on the HAM10000 test set under non-IID partitioning: (**a**) small-scale federation (K=5) and (**b**) large-scale federation (K=10).

The aggregated normalized confusion matrix displayed in Figure 8a further demonstrates the efficacy of the converged model. The confusion matrix is still highly diagonal despite the increased degree of data decentralization, suggesting that the learned feature representations maintain significant discriminative power across all lesion types. The recall values for each class vary from 90.00% to 96.37%, with NV having the highest recall at 96.37% and BKL at 92.40%. The recognition rates of the remaining classes—AKIEC (90.00%), BCC (90.20%), DF (90.18%), MEL (90.29%), and VASC (90.91%)—are very similar. While there is a little rise in inter-class confusion as compared to the K=5 situation, these mistakes are still quite small. The most obvious confusion made is with DF, which is often categorized as MEL (3.57%) and NV (5.36%). In a similar vein, VASC displays minor misclassification rates into DF, MEL, and NV (2.60% each), whilst MEL displays considerable confusion with NV (2.36%), VASC (2.08%), and BCC (1.80%). Although the corresponding misclassification rates are still rather low, AKIEC and BCC also show some confusion with other lesion classifications. These findings imply that the suggested methodology maintains balanced performance across categories and preserves relevant lesion-specific representations even when rising client fragmentation creates new categorization issues.

A quantitative comparison between the K=5 and K=10 configurations is provided in Table 4. The results indicate that the proposed federated framework achieves consistently high precision, recall, and F1-score values across all lesion classes under both settings. As expected, increasing the number of clients leads to modest reductions in performance for several categories, which can be attributed to the greater statistical heterogeneity and reduced amount of local training data available at each client. The suggested FedSwin-LHTP architecture shows the ability to scale well with more decentralization, as evidenced by the observed performance variations being very minimal. When combined, the convergence behavior, confusion matrix analysis, and class-wise evaluation metrics show that even when the federated environment gets significantly more difficult, the framework retains stable optimization and strong classification performance.

### 4.4. Results of ISIC2019 Dataset

#### 4.4.1. Scenario 1: K=5,α=0.5

The performance of the FL framework depicted in fig:pipeline1 was further assessed on a more extensive and diversified set of dermoscopic images using the ISIC2019 dataset. ISIC2019 has higher challenges than HAM10000 since it has more samples, more morphological variation between lesion categories, and a noticeable class imbalance. The dataset was divided across K=5 clients using a non-IID Dirichlet distribution with α=0.5 in order to examine the robustness of the suggested method in heterogeneous federated conditions. The combination of the Swin-T backbone and the LHTP mechanism showed steady optimization behavior throughout the training period, despite the statistical heterogeneity brought forth by this partitioning technique.

**Figure 9:**
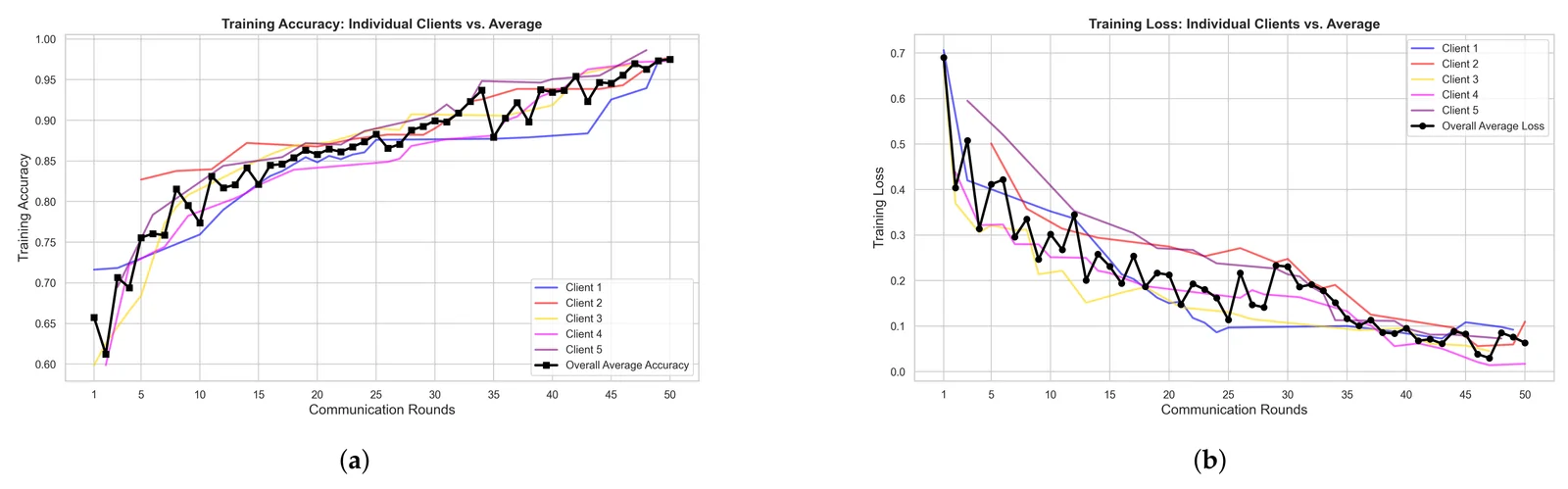
Local training accuracy and Loss plots for K=5 clients across federated learning rounds for the ISIC dataset with α=0.5. (**a**) Training Accuracy; (**b**) Training Loss.

Figure 9a,b show how client-wise training performance evolved throughout 50 communication stages. The uneven distribution of lesion categories among local datasets is reflected in the training trajectories, which show discernible disparities across participating clients during the early phases of learning. In contrast to Clients 1 and 4, which show higher loss values in the early stages of training, with approximate initial losses of 0.71 and 0.40, respectively, and Client 4 reaching nearly 0.55 around the sixth communication round, Client 3 maintains relatively low loss values from the initial communication rounds. In non-IID federated situations, where each client is exposed to unique data features and differing degrees of classification complexity, such differences are expected. However, as training goes on, the client trajectories eventually converge and the loss values steadily decline, suggesting that the federated optimization process effectively aggregates information. Despite the diverse data distribution, the related accuracy curves show a similar trend of progressive improvement across all clients, indicating that the framework is still able to train stable and transferable feature representations.

The aggregated normalized confusion matrix shown in Figure 11a provides further insight into the convergence model’s classification performance. Strong diagonal dominance is seen across the matrix, suggesting that most samples are appropriately classified into the appropriate lesion groups. Class-wise recall statistics show very constant recognition performance across all classes, ranging from 89.99% to 96.07%. NV has the highest recall (96.07%), followed by MEL (95.25%), SCC (94.25%), and BKL (92.89%), while BCC has the lowest recall (89.99%). Several significant confusion patterns are seen within physically comparable lesion types, despite the overall classification performance being good. BCC is typically confused with BKL (3.29%) and AKIEC (2.80%), whereas AKIEC is most commonly misclassified as BKL (3.92%) and DF (2.94%). In similar fashion, DF exhibits the most off-diagonal errors toward MEL (2.65%) and NV (5.29%), indicating some overlap in the learned feature representations for these categories. While VASC shows little confusion with NV (3.45%) and BKL (1.97%), SCC is occasionally mistaken with NV (1.98%) and DF (1.98%). With relatively slight misclassification rates into BCC (1.55%) and NV (1.89%), the MEL class notably obtains a recall of 95.25%, suggesting that malignant lesions are typically well recognized from the other classes.

When analyzed together, the class-wise performance shown in Figure 11a and the convergence behavior seen in Figure 9a,b indicate that the suggested FedSwin-LHTP framework can sustain stable optimization and robust classification performance on the more difficult ISIC2019 dataset. The prevalence of high diagonal values in the confusion matrix suggests that the discriminative information required for lesion detection is mostly conserved during the token pruning process, even though some confusion still exists across lesion categories with comparable visual features. These findings demonstrate that even when applied to a more extensive and varied skin-lesion classification job, the suggested architecture maintains its efficacy under heterogeneous federated learning situations.

#### 4.4.2. Scenario 2: K=10,α=0.5

Experiments were carried out on the ISIC2019 dataset employing K=10 clients under the same non-IID partitioning configuration (α=0.5) in order to further examine the scalability of the suggested FedSwin-LHTP architecture. A higher level of data fragmentation and statistical heterogeneity is introduced when the number of participating clients increases, leading to more varied local data distributions and possibly less steady local updates. These circumstances often increase the diversity across local models and decrease the quantity of data accessible at each client, making federated optimization more difficult. Nevertheless, during the training period, the suggested framework maintained good classification performance and continued to show robust convergence behavior.

**Figure 10:**
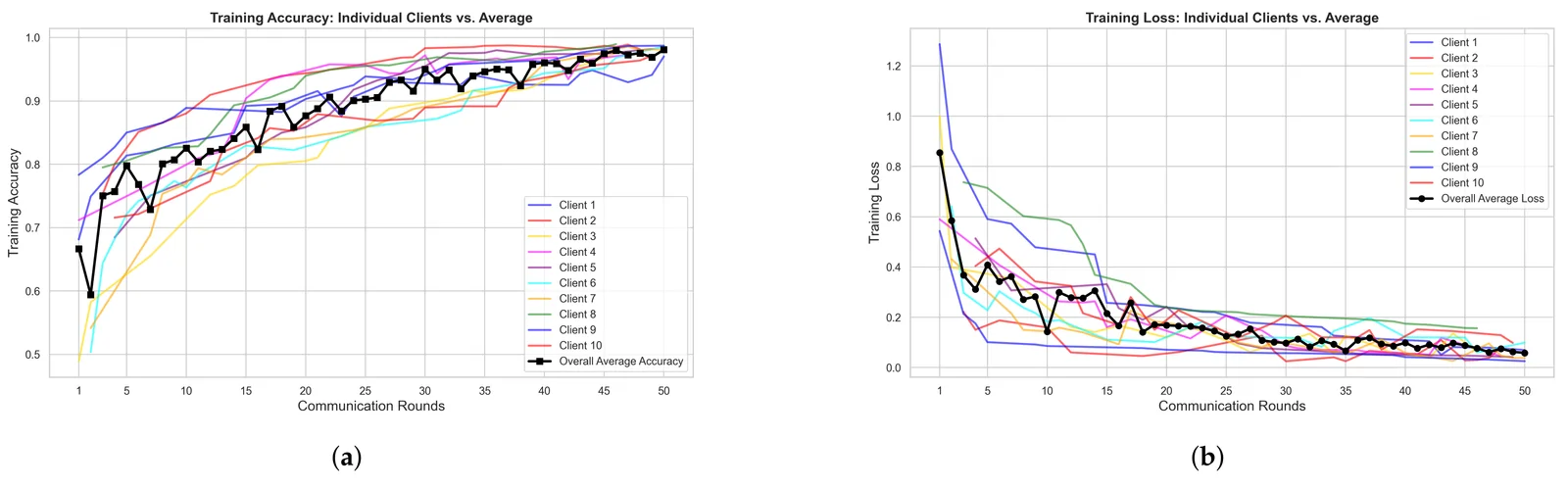
Local training accuracy and Loss plots for K=10 clients across federated learning rounds for the ISIC dataset with α=0.5. (**a**) Training Accuracy; (**b**) Training Loss.

Figure 10a,b show the training patterns demonstrating how client performance changed throughout 50 communication rounds. During the early phases of learning, the training accuracy rises quickly, demonstrating efficient information transfer via federated aggregation. While there are brief variations around communication rounds 6 and 39, which are probably caused by heterogeneous client updates and different local data properties, these variations do not last over time. Rather, the global model bounces back fast and keeps moving in the direction of convergence. The corresponding training loss stabilizes around 0.02 by the latter communication rounds, while the average training accuracy reaches around 98.5%. Despite the increasing decentralization brought about by the greater client population, the overall trends seen in the accuracy and loss curves indicate that the optimization process is still steady.

**Figure 11:**
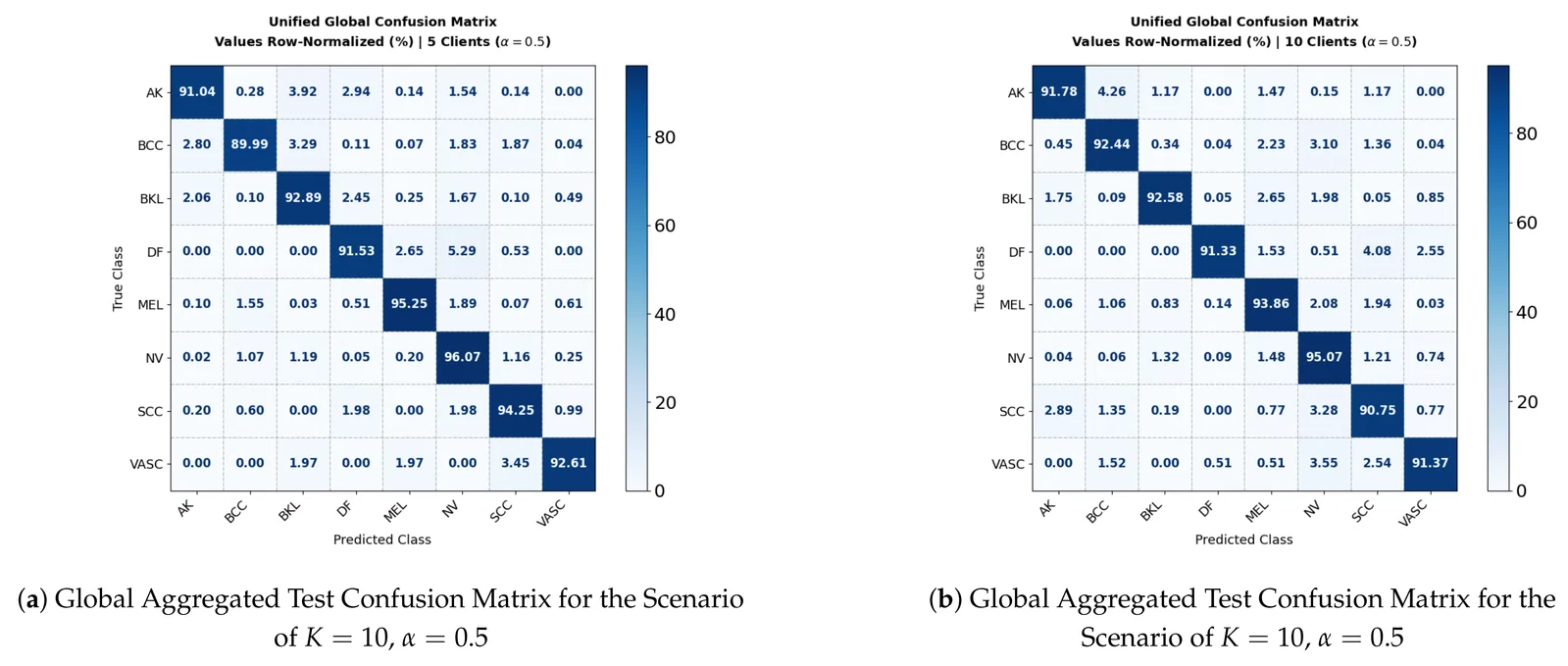
Global confusion matrices of the federated model on the ISIC2019 testset under non-IID partitioning: (**a**) small-scale federation (K=5) and (**b**) large-scale federation (K=10).

The aggregated normalized confusion matrix displayed in Figure 11b is incorporated to further examine the convergence model’s classification performance. The matrix shows high diagonal dominance, which is similar to the K=5 setup and shows that the majority of samples are appropriately classified to their corresponding lesion categories. The recall values for each class vary from 90.75% to 95.07%, with NV having the greatest recall of 95.07%, followed by MEL (93.86%), BKL (92.58%), and BCC (92.44%). While their identification rates are nevertheless competitive, SCC (90.75%) and VASC (91.37%) have relatively lower recall values. Several significant confusion patterns across lesion groups with overlapping visual features are revealed by examining the off-diagonal parts. While BCC is most commonly confused with NV (3.10%) and MEL (2.23%), AKIEC is most commonly misclassified as BCC (4.26%) and MEL (1.47%). While DF exhibits significant confusion with SCC (4.08%) and VASC (2.55%), MEL is occasionally misclassified as NV (2.08%) and SCC (1.94%). Similar to this, SCC is mistakenly classified as NV (3.28%) and AKIEC (2.89%), although VASC exhibits significant confusion with NV (3.55%) and SCC (2.54%). Despite these errors, the melanoma class maintains a recall of 93.86%, indicating that the framework is generally successful in distinguishing clinically important malignant lesions from other lesion classes. Overall, higher data partitioning has no effect on the discriminative representations of the federated Swin-T model, as seen by the prevalence of high diagonal values and the extremely tiny number of off-diagonal mistakes.

Table 5 provides a quantitative comparison of the K=5 and K=10 settings. The findings show comparable class-wise performance in both federated contexts, with all lesion categories maintaining strong accuracy, recall, and F1-scores. As anticipated, increasing the number of clients from five to ten results in a little decline in performance for a number of classes. This is due to the higher statistical heterogeneity and decreased local data availability that come with the more dispersed arrangement. Nonetheless, the total variations are still rather minor, suggesting that the suggested structure adapts well to growing clientele. When considered collectively, the convergence analysis, confusion matrix evaluation, and class-wise performance metrics indicate that even under difficult decentralized learning settings, the FedSwin-LHTP architecture retains strong optimization and classification capabilities on the ISIC2019 dataset.

### 4.5. Discriminative Performance Evaluation ViA AUC

**Figure 12:**
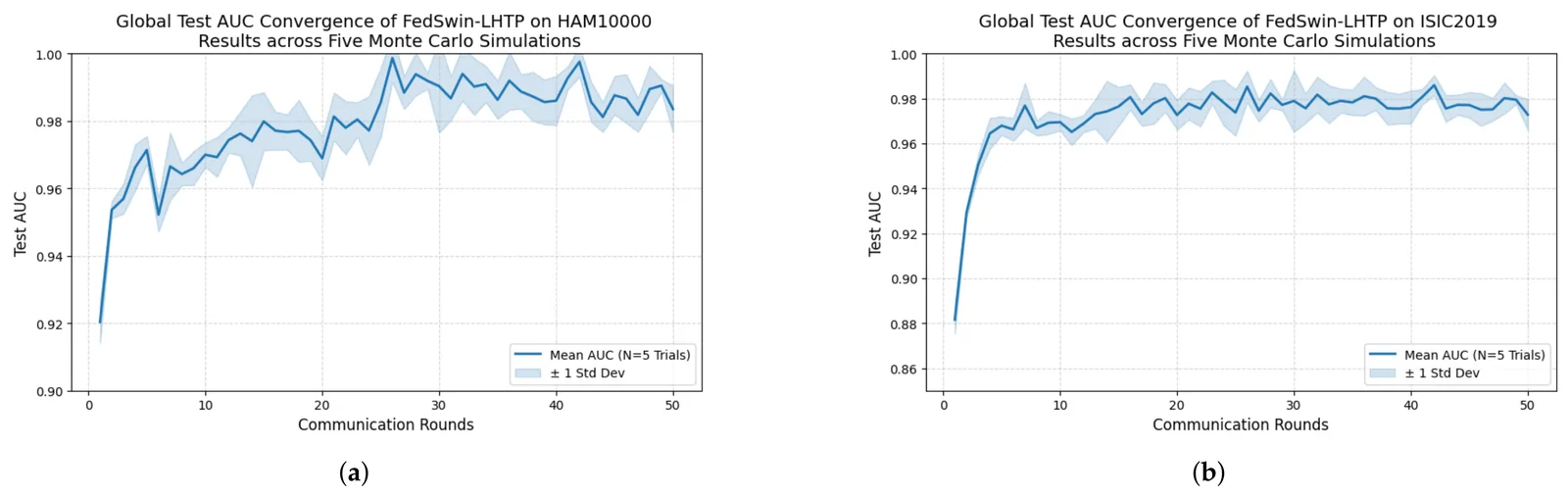
FedSwin-LHTP Performance Stability: Mean Global Test AUC (±SD). (**a**) Global Aggregated Test AUC—HAM10000 Dataset; (**b**) Global Aggregated Test AUC—ISIC2019 Dataset.

The Area Under the Receiver Operating Characteristic Curve (AUC-ROC), a crucial statistic for assessing diagnostic performance across different classification thresholds, was used to evaluate the discriminative power of the suggested FedSwin-LHTP architecture. We performed N=5 Monte Carlo simulations to verify the consistency and dependability of our methodology because the HAM10000 and ISIC2019 datasets are inherently non-IID in a federated setting. The global model’s convergence behavior throughout the communication rounds for the K=5 and K=10 client setups is shown in Figure 12a,b. These graphs’ shaded areas show the ±1 standard deviation (SD) for each of the five separate simulation runs. Despite the significant decrease in computing cost, this statistical rigor shows that the LHTP mechanism maintains strong diagnostic stability. In particular, the AUC curves show steady performance plateaus after fast convergence during the first communication cycles. Even in the more challenging large-scale federation scenario (K=10), the FedSwin-LHTP model maintains a high AUC, indicating that the structure-aware token pruning effectively preserves the spatial attention links required for distinguishing skin lesion features. In order to ensure that the efficiency savings obtained by token pruning do not come at the expense of diagnostic reliability, the low variation (narrow SD band) throughout the Monte Carlo trials demonstrates that the LHTP mechanism does not introduce considerable stochastic volatility into the global model. These results validate FedSwin-LHTP as a robust solution for privacy-preserving, resource-constrained medical imaging tasks. However, the precision for the DF and VASC categories is relatively on the lower side though the overall classification performance is strong. The low precision of these minority categories can be explained by severe class imbalance and morphological overlap of rare lesion types. As the data for DF and VASC only accounts for a small fraction of the total training samples, the model has less chance to learn highly discriminative representations for these classes, which in turn leads to more false-positive predictions. Similar observations have been reported in previous studies on dermoscopic image classification where rare lesion categories are especially difficult to distinguish under highly imbalanced data distributions.

### 4.6. Ablation Study

**Figure 13:**
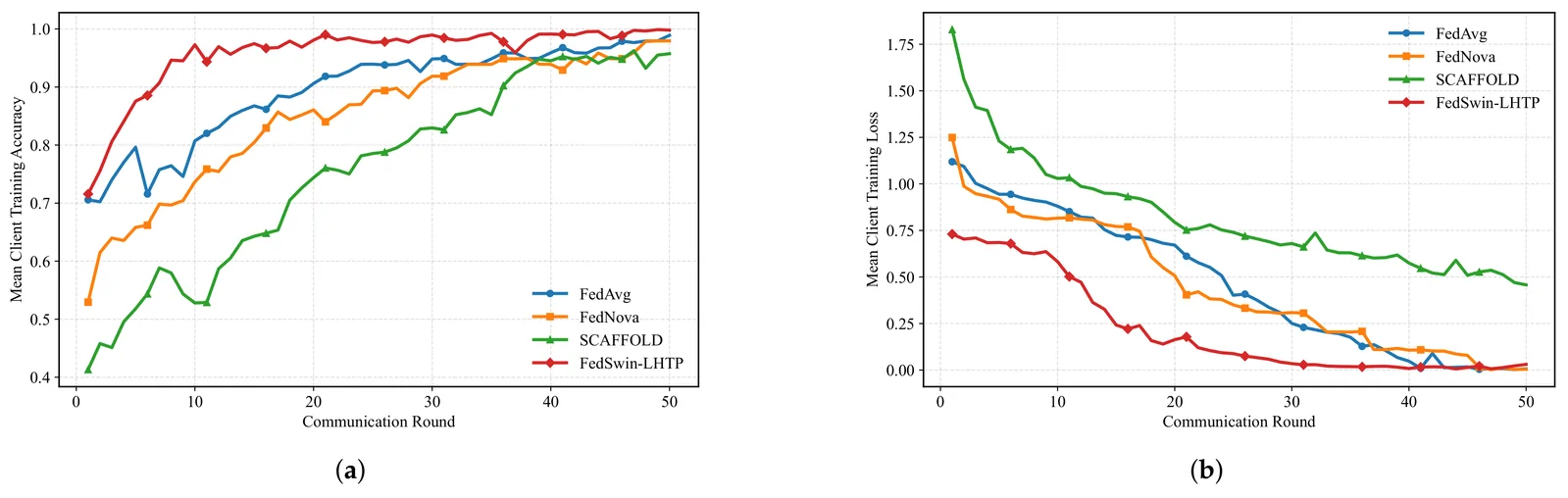
Average training accuracy and loss of K=5 clients for different federated aggregation methods on HAM10000 dataaset, α=0.5. (**a**) Average training accuracy; (**b**) Average training loss.

Figure 13 provides an ablation evaluation comparing the proposed FedSwin-LHTP framework against standard federated aggregation methodologies such as *FedAvg*, *FedNova*, and *SCAFFOLD* using the average performance across K=5 clients on the HAM10000 dataset. As demonstrated in the training accuracy trajectories, FedSwin-LHTP starts from a stronger initial state and converges rapidly within early communication rounds, maintaining a decisive advantage over all alternative aggregation techniques. This efficiency is further validated by the training loss dynamics, where FedSwin-LHTP achieves a steep, continuous drop toward minimal loss levels substantially faster than the baselines. In contrast, conventional aggregation methods exhibit significantly slower convergence profiles, with SCAFFOLD struggling to minimize training loss as effectively as FedNova and FedAvg. Overall, this comparative study confirms that the aggregation strategy embedded in FedSwin-LHTP provides superior optimization stability, enhanced sample efficiency, and faster client-side convergence under heterogeneous federated settings.

**Figure 14:**
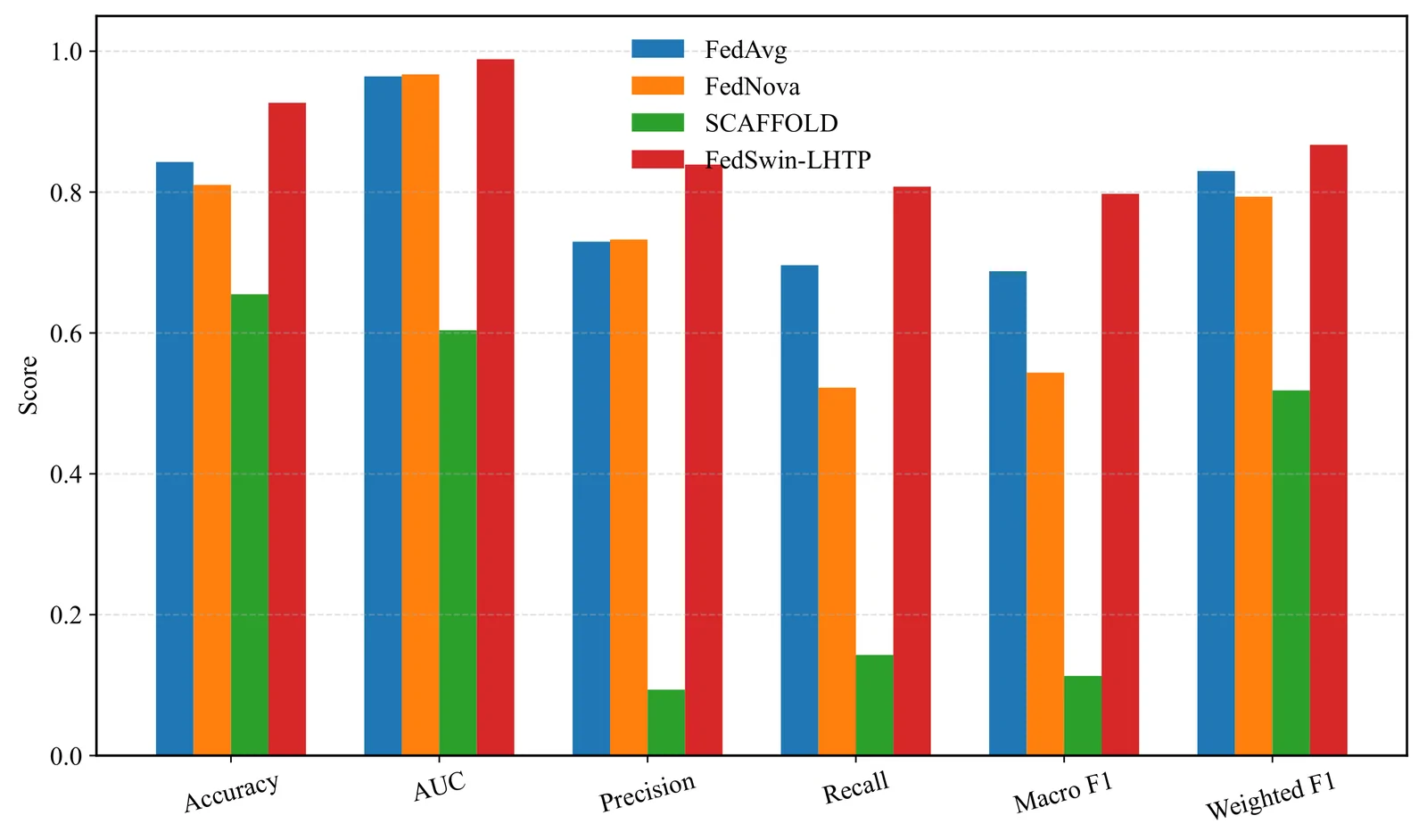
Test Peformance Comparison of Proposed FedSwin-LHTP and other FL aggregation baselines on HAM10000 dataset with K=5 clients and α=0.5.

The test performance comparison of proposed FedSwin-LHTP and FL aggregation baseline methods is presented in Figure 14. FedSwin-LHTP consistently outperforms all baseline aggregation strategies, achieving near-optimal performance across Accuracy, AUC, Precision, Recall, Macro F1, and Weighted F1. The baseline methods show lower performance scores across all evaluated metrics, with SCAFFOLD slightly outperforming FedNova and FedAvg.

**Figure 15:**
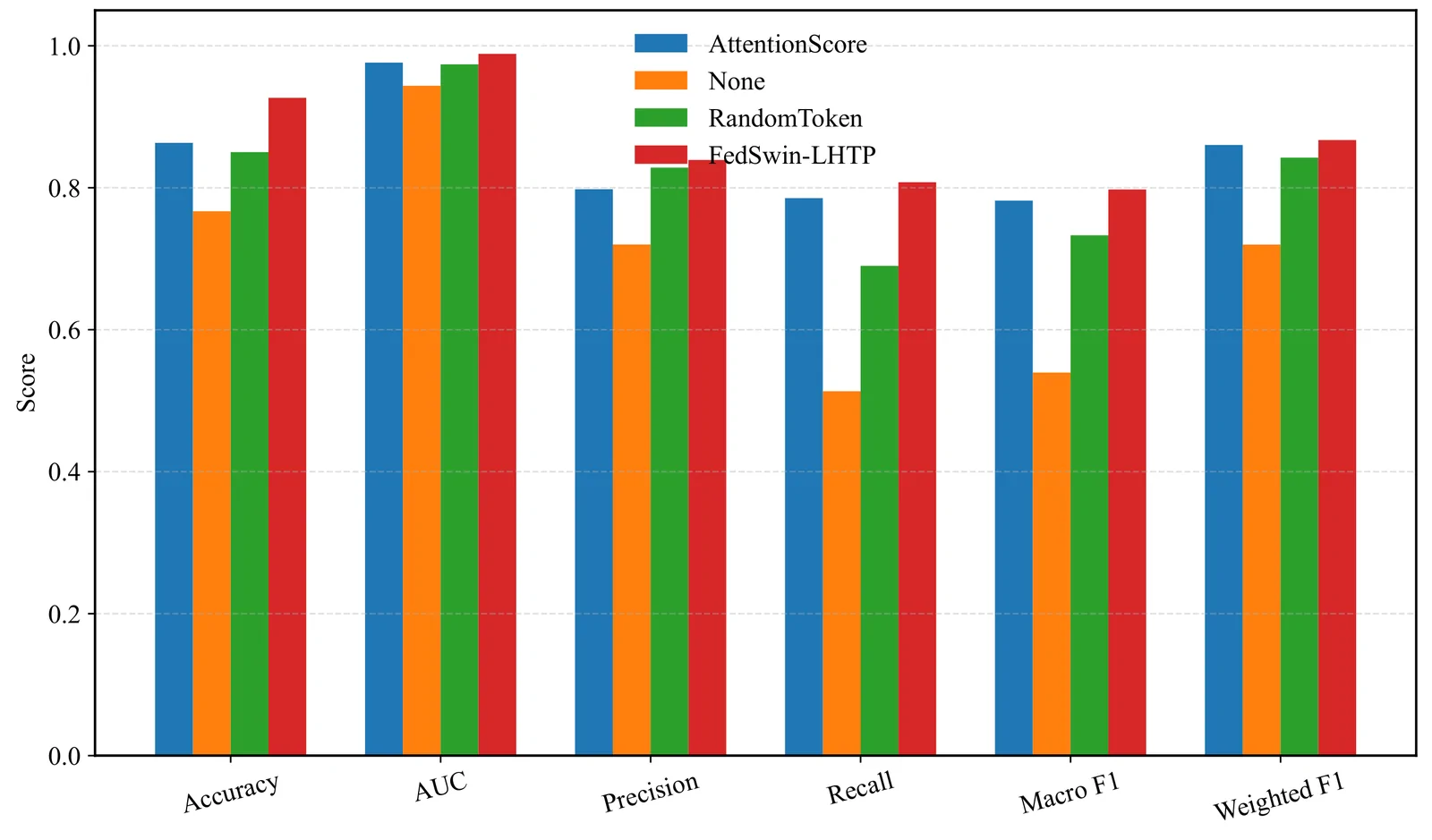
Performance Comparison of Proposed FedSkin-LHTP with several token pruning strategies on Ham10000 Dataset, K=5 clients and α=0.5.

To further analyze the effectiveness of the proposed approach, another ablation study compares the final classification performance across *K* = 5 clients on the HAM10000 dataset using FedSwin-LHTP and three token pruning strategies: AttentionScore-based pruning, Random pruning, and no pruning. As shown in Figure 15, across all metrics, omitting token pruning (*None*) severely degrades model performance—particularly in Recall and Macro F1—while heuristic strategies like *RandomToken* and *AttentionScore* yield moderate improvements. The proposed FedSwin-LHTP framework consistently achieves the highest scores across Accuracy, AUC, Precision, Recall, Macro F1, and Weighted F1, confirming that its targeted pruning mechanism optimizes feature extraction and client generalization under federated settings.

**Figure 16:**
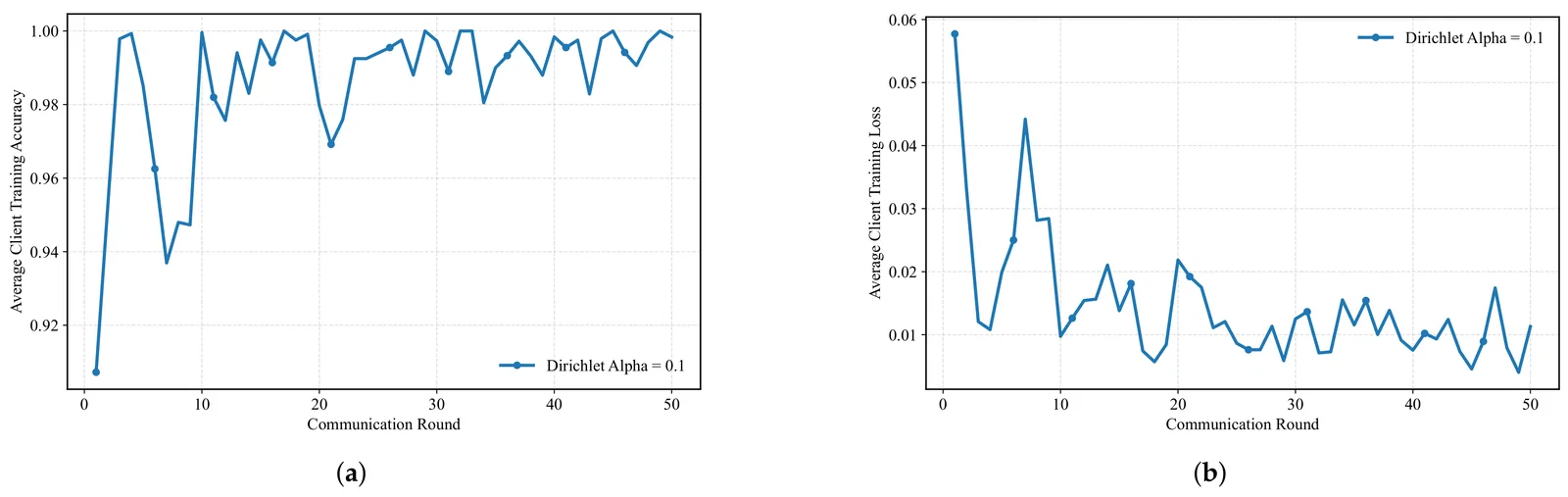
Average training accuracy and loss of K=5 clients for HAM10000 dataaset and α=0.1. (**a**) Average training accuracy; (**b**) Average training loss.

In Figure 16a, the average training accuracy and loss are plotted as a function of FL rounds for K=5 clients under extreme non-IID data distribution setting with α=0.1. In this situation, the average client training accuracy trajectory exhibits noticeable periodic fluctuations throughout the 50 communication rounds. These oscillations are primarily driven by local client drift and severe label skew across client updates. However, despite these minor perturbations, the framework rapidly converges within the first 10 rounds and keeps all accuracy fluctuations tightly bounded above 0.98, proving that the proposed approach effectively maintains global optimization stability under heavy client data heterogeneity. As observed in the training loss curve, the framework exhibits a sharp initial drop followed by pronounced periodic spikes in early rounds, directly mirroring the minor dips seen in the training accuracy profile. Together, these complementary curves confirm that the proposed approach maintains high optimization stability and effectively counters client-side variance, even under severe non-IID conditions.

### 4.7. Comparison with Existing Federated Learning Frameworks

The proposed FedSwin-LHTP architecture is compared with existing FL-based skin lesion classification techniques using CNN and ViT backbones in Table 6. While transformer-based techniques like Federated ViT [6] and FedViTBloc [28] reported accuracies of up to 94.1%, CNN-based techniques like FL-CNN [15] and SkinFLNet [23] achieved accuracies of 94.0% and 92.08%, respectively. Similarly, under various experimental conditions, the customized FedLSC method [43] and the federated DCNN framework of [20] obtained about 95% classification performance. However, differences in datasets, client setups, and data partitioning techniques make direct comparison based only on accuracy difficult. Additionally, a number of current approaches either use computationally demanding transformer structures or add complexity through privacy-preserving and customization features.

On the other hand, the suggested FedSwin-LHTP framework uses the Lightweight Hessian-Inspired Token Pruning method to reduce computational complexity while achieving a classification accuracy of 96.1%. The approach successfully balances diagnostic performance and computational efficiency by dynamically suppressing about 60% of the Stage-1 tokens, which makes it ideal for resource-constrained and diverse federated healthcare contexts. Additionally, its privacy-preserving collaborative learning paradigm makes it possible for several healthcare facilities to work together to create reliable skin lesion classification models without disclosing private patient information. This promotes safe multi-institutional cooperation and supports scalable computer-aided skin cancer diagnosis. All of these findings show that FedSwin-LHTP’s main benefit is not only its competitive classification performance but also its enhanced trade-off between accuracy, computational economy, and practical deployability in actual clinical situations.

### 4.8. Statistical Analysis of Adaptive Token Pruning

**Figure 17:**
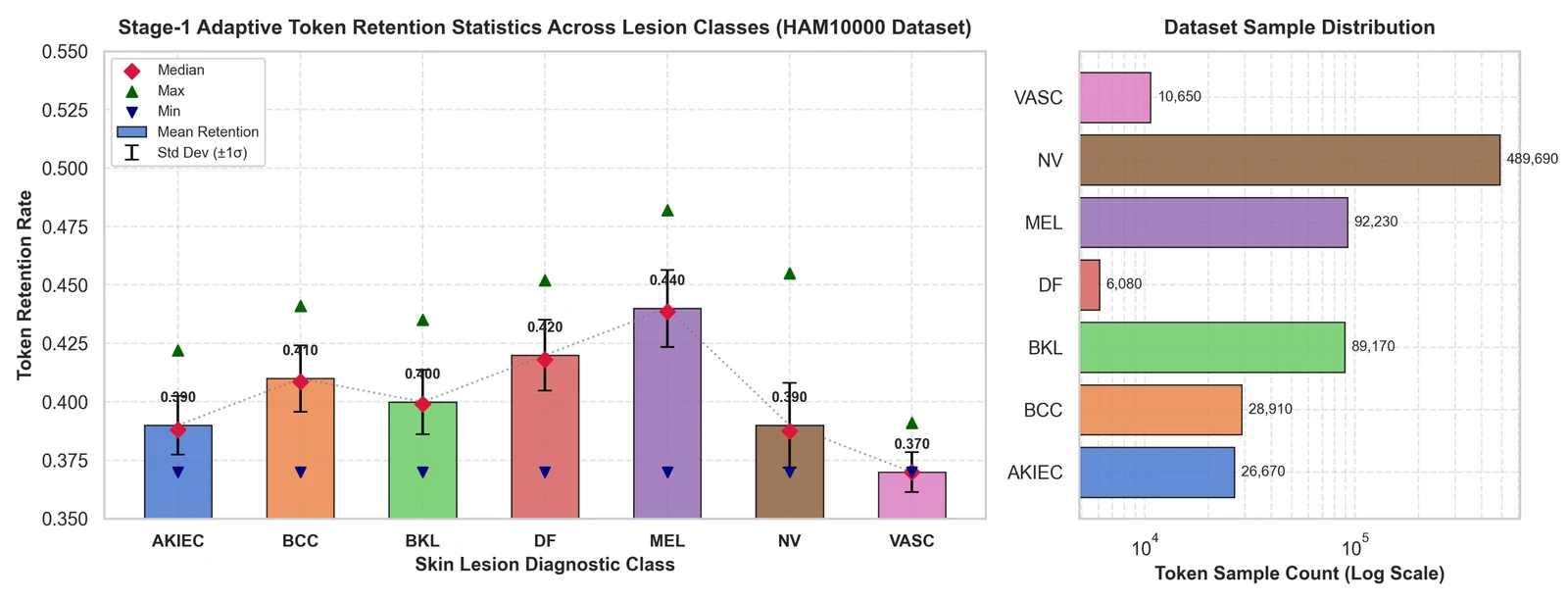
Stage-1 adaptive token retention statistics across lesion classes.

**Figure 18:**
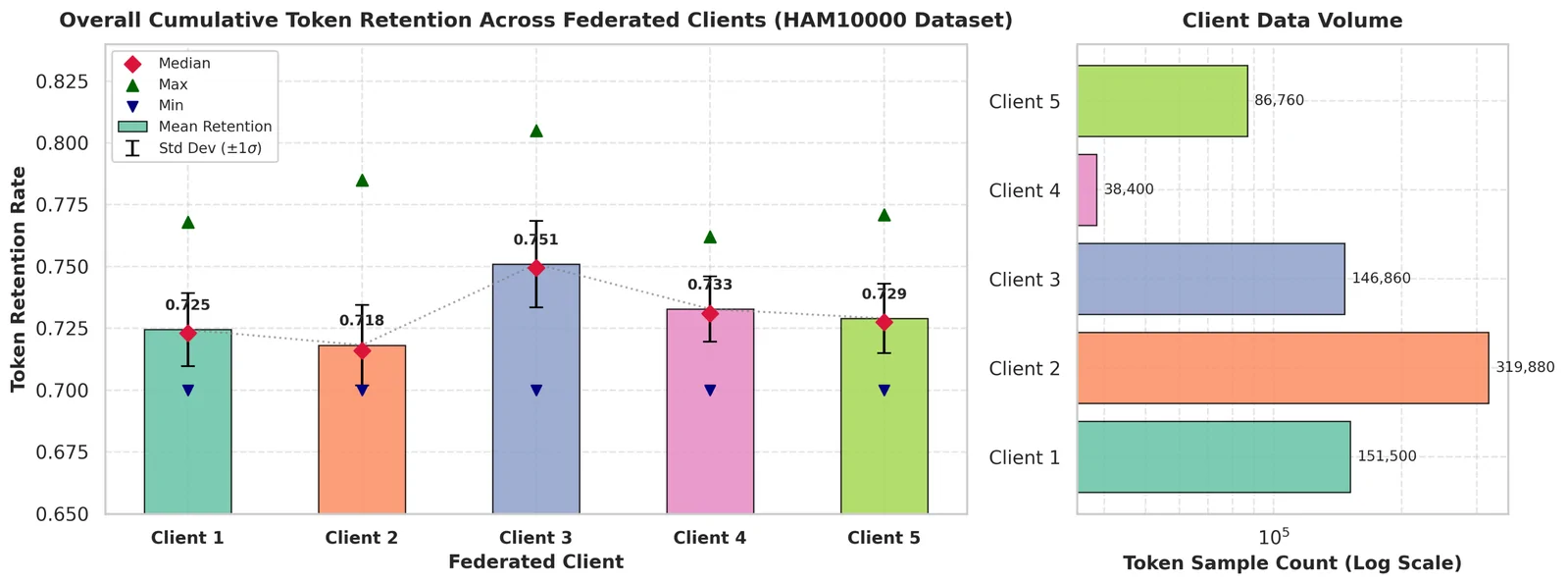
Overall cumulative token retention across federated clients.

A statistical investigation of the adaptive pruning mechanism was carried out across lesion classes of the HAM10000 dataset and federated clients in order to better examine the behavior of the suggested LHTP technique. The class-wise analysis reports the Stage-1 adaptive token retention ratio immediately after LHTP pruning as shown in Figure 17, whereas, Figure 18 presents the client-wise analysis for the overall cumulative token retention across the hierarchical Swin Transformer pipeline. The suggested LHTP module produces data-dependent token retention rates by dynamically determining token relevance depending on the local optimization landscape, in contrast to traditional methods that employ a preset pruning ratio.

The class-wise token retention statistics are shown in Figure 17, where each lesion category’s mean, median, minimum, maximum, and standard deviation of the retained token ratios are given. The actual retention ratio differs among lesion classes, indicating the adaptability of the suggested pruning technique, even though the goal average retention is about 40%. Melanoma has the highest average token retention (around 44%), suggesting that morphologically complex lesions maintain more information patch tokens. Melanocytic nevus and vascular lesions, on the other hand, retain fewer tokens (about 37% and 39%, respectively), indicating that superfluous visual information may be eliminated more forcefully without affecting lesion representation. Further evidence that the adaptive threshold stays constant while reacting to class-specific morphological traits comes from the very small standard deviation across all categories. Figure 17 additionally illustrates the corresponding dataset sample distribution, highlighting that the observed retention behavior is not solely determined by class frequency but also by the intrinsic visual complexity of each lesion category.

To evaluate the robustness of the adaptive threshold under heterogeneous federated environments, Figure 18 summarizes the token retention statistics across the participating clients. Despite the non-IID data distributions and varying local dataset sizes, the average retention ratio remains highly consistent across all clients, ranging from approximately 71.8% to 75.1%, with only small standard deviations. This demonstrates that the proposed adaptive pruning mechanism produces stable token selection regardless of client-specific data heterogeneity. Figure 18 further presents the distribution of training samples across clients, confirming substantial variations in local data volume while maintaining consistent pruning behavior. The class-wise analysis reports the proportion of tokens retained after the adaptive pruning stage, whereas the client-wise statistics correspond to the cumulative token retention across the hierarchical Swin Transformer pipeline.

Overall, these results demonstrate that the proposed LHTP module performs dynamic and data-dependent token selection rather than enforcing a fixed pruning ratio. The low variance observed across lesion classes and federated clients indicates that the adaptive threshold generalizes well to heterogeneous clinical data while consistently preserving diagnostically relevant token representations and eliminating redundant tokens.

### 4.9. Complexity Analysis

#### 4.9.1. Computational Complexity

The computational efficiency of the proposed FedSwin-LHTP framework arises from the combination of window-based self-attention and adaptive token pruning. Let *N* denote the number of input tokens and *d* represent the embedding dimension.

A conventional ViT computes the global self-attention using pairwise interactions among all tokens [44], resulting in a computational complexity of $O(N^{2}d)$.

For the Swin Transformer backbone employed in this work, the Stage-1 feature map contains

N=56×56=3136

tokens. Consequently, global self-attention requires

$$N^{2}=3136^{2}=9,834,496$$

pairwise token interactions. Unlike conventional ViTs, the Swin Transformer restricts self-attention computation to local windows of size M×M, where M=7. Each window therefore contains

$$M^{2}=49$$

tokens and the total number of windows becomes

$$W=\frac{N}{M^{2}}=\frac{3136}{49}=64.$$

The computational complexity of window-based self-attention is therefore $O(NM^{2}d)$, which scales linearly with the number of tokens.

After Stage 1, the proposed LHTP module is applied for dynamic selection of diagnostically important tokens. Experimental evaluations indicate that approximately 40% of Stage-1 tokens are retained on average, corresponding to an effective pruning ratio of approximately (60%). Therefore,

K≈0.4N≈1254.

The computational complexity of subsequent transformer stages becomes $O(KM^{2}d)$, where (K<N). Relative to the original token set, the computational workload of attention operations in deeper transformer stages is reduced by approximately

$$1-\frac{K}{N}=1-\frac{1254}{3136}\approx 60\%.$$

Furthermore, the complexity of computing token-importance scores is O(NC), where *C* denotes the token embedding dimension. Since this operation grows linearly with the number of tokens and reuses gradient information already available during backpropagation, its computational overhead is negligible compared with self-attention operations.

Consequently, the proposed FedSwin-LHTP framework reduces the computational burden of local training and inference by suppressing redundant tokens while largely preserving diagnostically relevant lesion representations.

#### 4.9.2. Communication Complexity

Let *P* denote the number of trainable model parameters and $K_{c}$ represent the number of participating clients in each communication round. During federated learning, each selected client downloads the global model parameters from the server and uploads its locally updated model parameters after training. Therefore, the communication cost per federated round is

$$O\left(2K_{c}P\right)=OK_{c}P$$

where the first term corresponds to model distribution and the second term corresponds to model aggregation.

The proposed LHTP module operates entirely on local token representations and does not introduce additional trainable parameters or auxiliary communication messages. Consequently, the communication complexity remains identical to that of the baseline FedProx-based Swin Transformer framework. Therefore, the proposed method achieves computational savings at the client side without increasing communication overhead, thereby maintaining the scalability and privacy-preserving characteristics of federated learning.

## 5. Discussion

As demonstrated by the experimental results, the proposed *FedSwin-LHTP* framework achieves an effective balance between computational efficiency and predictive performance for heterogeneous federated skin lesion classification. This performance arises from the complementary strengths of three core components: the *Swin Transformer*, which learns hierarchical local and global representations through shifted window-based self-attention; the LHTP module, which adaptively removes redundant tokens while preserving diagnostically relevant information; and the *FedProx* optimization strategy, which mitigates client drift by constraining local model updates under non-IID data distributions. Together, these components enable stable federated optimization and robust generalization across heterogeneous clinical institutions.

On the HAM10000 dataset, the proposed framework converged smoothly under both client configurations, exhibiting a steady reduction in client-to-client variability while maintaining strongly diagonal confusion matrices. With K=5 clients, the framework achieved 96.12% accuracy and a 96.3% weighted F1-score, whereas increasing the federation to K=10 clients resulted in a slight reduction to 94.58% accuracy and a 94.9% weighted F1-score. The superior performance with fewer clients can be attributed to the availability of more local training samples per institution, resulting in more reliable local updates and improved global convergence. In contrast, increasing the number of clients reduces the local data available at each institution and amplifies statistical heterogeneity, leading to greater variability among client updates and making global aggregation more challenging. Nevertheless, the modest performance degradation demonstrates that the proposed framework scales effectively to increasingly decentralized healthcare environments while maintaining stable optimization and high classification performance. The class-wise results further indicate that common lesion categories such as NV and MEL are consistently recognized, whereas minority classes including DF and VASC remain more challenging because of severe class imbalance and visual similarity. In this work, Weighted Cross-Entropy Loss was adopted as a stable and computationally efficient strategy for mitigating class imbalance under heterogeneous federated settings. Although more advanced imbalance-aware approaches, such as focal loss, class-balanced loss, and federated oversampling, may further improve the recognition of underrepresented lesion categories, their investigation is beyond the scope of the present study and represents an important direction for future research.

A similar trend was observed on the ISIC2019 dataset, which presents a more challenging benchmark because of its larger scale, stronger class imbalance, and greater morphological diversity. With K=5 clients, the framework achieved 96.41% accuracy and a 96.5% weighted F1-score, while increasing the federation to K=10 clients resulted in a slight decrease to 94.01% accuracy and a 94.2% weighted F1-score. Despite the increased optimization difficulty, the confusion matrices retained strong diagonal dominance, indicating robust classification performance. Most misclassifications occurred among visually similar or underrepresented lesion classes, including DF, VASC, SCC, and BCC, whereas melanoma maintained consistently high recognition performance.

The robustness of the proposed framework is further supported by the Monte Carlo analysis. Across five independent trials with different random initializations, the model consistently converged with narrow confidence intervals and low performance variability, indicating that the integration of LHTP does not introduce optimization instability during federated training. The accompanying AUC analysis further confirms the framework’s reliable discriminative capability across lesion categories. From a practical perspective, the combination of the Swin Transformer, structure-aware LHTP, and FedProx enables efficient learning under heterogeneous non-IID settings by preserving diagnostically relevant lesion representations, reducing redundant computation, and stabilizing federated optimization.

Although the proposed framework preserves patient privacy by ensuring that raw clinical data remain at participating institutions and only model parameters are exchanged, it follows the standard Federated Learning privacy assumption and does not explicitly address gradient leakage or model inversion attacks. Furthermore, while the LHTP module substantially reduces computational complexit by pruning redundant tokens at an early stage of the Swin Transformer, this study did not quantify peak memory footprint or energy consumption on resource-constrained clinical edge devices. These limitations motivate future investigations into stronger privacy-preserving mechanisms, such as Differential Privacy and Secure Aggregation, formal evaluation against gradient reconstruction attacks, and hardware-level profiling on representative clinical edge platforms.

## 6. Conclusions

This paper presented *FedSwin-LHTP*, a privacy-preserving federated learning framework for automated skin lesion classification that integrates a Swin Transformer backbone with an LHTP strategy and FedProx optimization. By adaptively pruning redundant tokens at the first stage of the Swin Transformer, the proposed framework reduces computational and memory requirements before subsequent attention operations while preserving diagnostically relevant representations. Experimental evaluation on the HAM10000 and ISIC2019 datasets under five- and ten-client federated settings demonstrated stable convergence and global test accuracies exceeding 94%, confirming the effectiveness and robustness of the proposed framework under heterogeneous non-IID data distributions.

Overall, the results demonstrate that the combination of the Swin Transformer, structure-aware LHTP, and FedProx enables efficient, scalable, and privacy-preserving federated learning without compromising diagnostic performance. Although validated on skin lesion classification, the proposed framework is readily applicable to other privacy-sensitive medical imaging applications, including retinal disease diagnosis, histopathology, chest X-ray analysis, CT-based disease detection, and brain MRI classification. Future work will focus on evaluating the framework in larger federated deployments, integrating stronger privacy-preserving mechanisms such as Secure Aggregation and Differential Privacy, investigating advanced imbalance-aware federated learning strategies for improved recognition of minority lesion classes, performing hardware-level evaluation of memory usage and energy efficiency on clinical edge devices, and extending the proposed framework to a broader range of medical imaging and clinical diagnostic applications.

## Figures and Tables

**Table 1:** Summary of Federated Learning Literature for Skin Lesion Classification.

Ref.	Model Backbone	Dataset Profile	Performance	Key Limitations
[15]	Custom CNN + FedAvg	4-class custom dataset	Acc.: 94.00%	Small dataset; no advanced optimization; limited edge-device evaluation.
[16]	EfficientNetV2S, EfficientNetB3, ResNet50, MobileNetV2, NasNetMobile	Downsampled dermoscopy dataset	Acc.: 90.64% (Non-IID), 89.83% (IID)	Information loss due to downsampling; computational trade-offs.
[17]	FedPerl semi-supervised framework	71k images from five datasets	Dice: 0.812, AUC: 0.935	High computation cost; class imbalance sensitivity; no privacy guarantees.
[18]	CNN + hybrid feature selection	HAM10000, ISIC	Acc.: 94.80%	CNN-only features; limited real-world FL validation.
[19]	FedMix aggregation framework	Multi-institutional dermatology images	Dice: 0.843	High local training overhead; unstable under severe imbalance.
[20]	Deep CNN + FedAvg	ISIC, PH2, multi-source datasets	Acc.: 98.00%, AUC: 0.951	Limited cross-population and deployment evaluation.
[21]	PMM-FL multimodal framework	Multimodal ISIC cohort	Acc.: 92.32%, AUC: 0.918	Requires complete multimodal records; complex aggregation.
[22]	HSSF semi-supervised FL	Skin lesion and polyp datasets	Acc.: 91.10%	Sensitive to pseudo-label quality; optimization complexity.
[23]	SkinFLNet (FL-VGG16)	ISBI 2016	Acc.: 92.08%	Limited FL settings and dataset diversity.
[24]	Asynchronous FL	ISIC2019	Acc.: 89.25%	Reduced representation capacity; communication dependency.
[25]	DenseNet201 + FedAvg	ISIC dataset	Acc.: 91.70%	Few simulated clients; limited heterogeneity assessment.
[26]	Med-VCD optimizer	Multi-task visual-language benchmarks	Hallucination calibration metrics	High computational and optimization overhead.
[27]	MedViTV2 + KAN	Corrupted medical datasets	Strong multiclass robustness	Complex architecture; extensive tuning required.
[28]	FedViTBloc (ViT + FHE + DP + Blockchain)	HAM10000	Acc.: 94.10%	High communication and latency overhead.
[29]	CFWL (CNN + VGG16)	ISIC dataset	Acc.: 90.00%	Large communication payload; scalability concerns.
[30]	ViT and CNN ensemble	Multi-class skin cancer dataset	Acc.: 92.14%	High computational cost; limited privacy considerations.
[31]	CNN with augmentation	7-class skin lesion dataset	Acc.: 93.25%	Limited multi-scale and long-range feature learning.

**Table 2:** Dataset Distribution Under Different Federated Learning Configurations.

Dataset	Total Images	FL Setup (K,α)	Training Set (80%)	Test Set (20%)
HAM10000	10,015	K=5,α=0.5	8012	2003
		K=10,α=0.5		
ISIC2019	25,331	K=5,α=0.5	20,265	5066
		K=10,α=0.5		

**Table 3:** Training Parameters of the Proposed FedSwin-LHTP Framework.

Parameter	Value	Parameter	Value
Backbone Model	Swin Transformer-Tiny	Framework	PyTorch 2.6.0.dev20241001
Optimizer	SGD	Learning Rate	$1\times 10^{-5}$
Loss Function	Weighted Cross Entropy	Federation Algorithm	FedProx
Total No. of Clients	5, 10	Client Participation Rate	40%
Local Epochs	3	Mini-Batch Size	8
Maximum FL Rounds	50	Weight Decay	$1\times 10^{-4}$
LHTP Position	After Stage 1	Pruning Strategy	Dynamic Token Pruning
Threshold Selection	Adaptive Score-Based	FedProx Coefficient (μ)	0.01
Importance Score	$∥\nabla L_{i}{∥}_{2}+\lambda \;\mathrm{Var}\left(\nabla L_{i}\right)$	Data Partitioning	Dirichlet Distribution
Dirichlet Parameter (α)	0.5	Threshold sensitivity parameter (β)	0.5

**Table 4:** Class-wise Performance Comparison (Mean ± SD) over 5 Monte Carlo Simulations on the HAM10000 Dataset.

Class	K=5 Clients			K=10 Clients		
	**Prec. (%)**	**Rec. (%)**	**F1 (%)**	**Prec. (%)**	**Rec. (%)**	**F1 (%)**
AKIEC	83.6 ± 1.2	91.8 ± 0.9	87.5 ± 0.7	86.2 ± 1.5	90.0 ± 1.1	88.0 ± 0.8
BCC	89.7 ± 0.8	90.8 ± 1.2	90.2 ± 0.5	74.2 ± 2.1	90.2 ± 1.0	81.4 ± 1.4
BKL	95.1 ± 0.5	94.0 ± 0.8	94.6 ± 0.4	96.3 ± 0.6	92.5 ± 0.9	94.4 ± 0.5
DF	62.6 ± 2.5	89.7 ± 1.5	73.7 ± 1.8	54.3 ± 3.0	90.2 ± 1.2	67.8 ± 2.0
MEL	95.1 ± 0.6	95.0 ± 0.7	95.0 ± 0.4	95.6 ± 0.8	90.3 ± 1.1	92.9 ± 0.7
NV	99.4 ± 0.2	97.4 ± 0.4	98.4 ± 0.2	99.7 ± 0.1	96.4 ± 0.5	98.0 ± 0.3
VASC	64.8 ± 1.8	95.9 ± 0.6	77.4 ± 1.2	63.6 ± 2.2	90.9 ± 1.3	74.9 ± 1.5
Macro	84.3 ± 0.9	93.5 ± 0.6	88.1 ± 0.5	81.4 ± 1.2	91.5 ± 0.8	85.3 ± 0.9
Weighted	96.6 ± 0.3	96.1 ± 0.3	96.3 ± 0.2	95.6 ± 0.4	94.6 ± 0.5	94.9 ± 0.4
Accuracy	96.12 ± 0.25%			94.58 ± 0.38%		

**Table 5:** Class-wise Performance Comparison (Mean ± SD) over 5 Monte Carlo Simulations on the ISIC2019 Dataset.

Class	K=5 Clients			K=10 Clients		
	**Prec. (%)**	**Rec. (%)**	**F1 (%)**	**Prec. (%)**	**Rec. (%)**	**F1 (%)**
AK	87.8 ± 1.1	93.8 ± 0.7	90.7 ± 0.6	89.2 ± 1.3	91.8 ± 0.9	90.5 ± 0.7
BCC	96.6 ± 0.5	96.4 ± 0.6	96.5 ± 0.3	94.6 ± 0.9	92.4 ± 1.1	93.5 ± 0.7
BKL	94.8 ± 0.4	95.1 ± 0.5	95.0 ± 0.3	94.4 ± 0.7	92.6 ± 0.8	93.5 ± 0.5
DF	61.5 ± 2.8	94.7 ± 1.2	74.6 ± 1.9	59.3 ± 3.2	91.3 ± 1.5	71.9 ± 2.2
MEL	98.2 ± 0.3	96.3 ± 0.4	97.3 ± 0.2	94.6 ± 1.0	93.9 ± 0.9	94.2 ± 0.6
NV	99.4 ± 0.2	97.0 ± 0.3	98.2 ± 0.2	97.9 ± 0.4	95.1 ± 0.6	96.5 ± 0.3
SCC	81.8 ± 1.5	95.5 ± 0.8	88.1 ± 0.9	66.6 ± 2.5	91.5 ± 1.2	77.1 ± 1.7
VASC	66.3 ± 2.1	94.1 ± 1.0	77.8 ± 1.4	59.6 ± 2.8	93.4 ± 1.1	72.7 ± 1.9
Macro	85.8 ± 0.9	95.4 ± 0.5	89.8 ± 0.6	82.0 ± 1.3	92.8 ± 0.9	86.2 ± 0.8
Weighted	96.9 ± 0.2	96.4 ± 0.2	96.5 ± 0.2	94.2 ± 0.5	94.0 ± 0.6	94.2 ± 0.4
Accuracy	96.41 ± 0.21%			94.01 ± 0.35%		

**Table 6:** Comparison of the proposed FedSwin-LHTP framework with recent federated skin lesion classification methods.

Reference	Method	Backbone	Dataset	Accuracy (%)
[6]	ViT + FedAvg	ViT-B/16	HAM10000, ISIC2019	90.0, 87.6
[15]	FL-CNN	Custom CNN	4-class skin dataset	94.0
[23]	FL-VGG16	VGG16	ISBI 2016	92.08
[28]	ViT + Blockchain + DP + FHE	ViT	HAM10000	94.1
[20]	Federated DCNN	Custom DCNN	ISIC, DermIS, PAD-UFES-20	95.1
[43]	Personalized FL	CNN	HAM10000	∼95.0
This work	FedSwin-LHTP	Swin-T + LHTP	HAM10000, ISIC2019	96.1

## Data Availability

The datasets used in this study are publicly available. The HAM10000 dataset is available from the Harvard Dataverse repository at https://dataverse.harvard.edu/dataset.xhtml?persistentId=doi:10.7910/DVN/DBW86T and the ISIC2019 dataset is available through the International Skin Imaging Collaboration (ISIC) archive at https://challenge.isic-archive.com/data/#2019.

## Abbreviations

The following abbreviations are used in this manuscript:

WCE	Weighted Cross-Entropy
non-IID	Non-Independent and Identically Distributed
LHTP	Lightweight Hessian-Inspired Token Pruning
ISIC	International Skin Imaging Collaboration
HAM10000	Human Against Machine with 10,000 Dermoscopic Images
MLP	Multi-Layer Perceptron
FedProx	Federated Proximal Optimization
FedAvg	Federated Averaging
ViT	Vision Transformer
